# Molecular characterization of colorectal adenomas reveals POFUT1 as a candidate driver of tumor progression

**DOI:** 10.1002/ijc.32627

**Published:** 2019-08-30

**Authors:** Malgorzata A. Komor, Meike de Wit, Jose van den Berg, Sanne R. Martens de Kemp, Pien M. Delis‐van Diemen, Anne S. Bolijn, Marianne Tijssen, Tim Schelfhorst, Sander R. Piersma, Davide Chiasserini, Joyce Sanders, Christian Rausch, Youri Hoogstrate, Andrew P. Stubbs, Mark de Jong, Guido Jenster, Beatriz Carvalho, Gerrit A. Meijer, Connie R. Jimenez, Remond J.A. Fijneman, In collaboration with the NGS‐ProToCol Consortium

**Affiliations:** ^1^ Department of Pathology Netherlands Cancer Institute Amsterdam The Netherlands; ^2^ Oncoproteomics Laboratory, Amsterdam UMC, Vrije Universiteit Amsterdam, Medical Oncology Amsterdam The Netherlands; ^3^ Department of Urology Erasmus Medical Center Rotterdam Rotterdam The Netherlands; ^4^ Department of Bioinformatics Erasmus Medical Center Rotterdam Rotterdam The Netherlands; ^5^ GenomeScan Leiden The Netherlands; ^6^ See Appendix for consortium members

**Keywords:** colorectal adenoma, adenoma‐to‐carcinoma progression, POFUT1

## Abstract

Removal of colorectal adenomas is an effective strategy to reduce colorectal cancer (CRC) mortality rates. However, as only a minority of adenomas progress to cancer, such strategies may lead to overtreatment. The present study aimed to characterize adenomas by in‐depth molecular profiling, to obtain insights into altered biology associated with the colorectal adenoma‐to‐carcinoma progression. We obtained low‐coverage whole genome sequencing, RNA sequencing and tandem mass spectrometry data for 30 CRCs, 30 adenomas and 18 normal adjacent colon samples. These data were used for DNA copy number aberrations profiling, differential expression, gene set enrichment and gene‐dosage effect analysis. Protein expression was independently validated by immunohistochemistry on tissue microarrays and in patient‐derived colorectal adenoma organoids. Stroma percentage was determined by digital image analysis of tissue sections. Twenty‐four out of 30 adenomas could be unambiguously classified as high risk (*n* = 9) or low risk (*n* = 15) of progressing to cancer, based on DNA copy number profiles. Biological processes more prevalent in high‐risk than low‐risk adenomas were related to proliferation, tumor microenvironment and Notch, Wnt, PI3K/AKT/mTOR and Hedgehog signaling, while metabolic processes and protein secretion were enriched in low‐risk adenomas. DNA copy number driven gene‐dosage effect in high‐risk adenomas and cancers was observed for *POFUT1*, *RPRD1B* and *EIF6*. Increased POFUT1 expression in high‐risk adenomas was validated in tissue samples and organoids. High POFUT1 expression was also associated with Notch signaling enrichment and with decreased goblet cells differentiation. In‐depth molecular characterization of colorectal adenomas revealed *POFUT1* and Notch signaling as potential drivers of tumor progression.

## Abstract 2

**What's new?**

Removal of colorectal adenomas is an effective strategy to reduce colorectal cancer (CRC) mortality rates. However, as only a minority of adenomas progress to cancer, such strategies may lead to overtreatment. While high‐risk adenomas, defined by specific DNA copy number aberrations, have an increased risk of progression, the mechanisms underlying colorectal adenoma‐to‐carcinoma progression remain unclear. This molecular characterization of colorectal adenomas, CRCs, and normal adjacent colon samples demonstrates that biological processes inherent to CRC are already more active in high‐risk adenomas compared to low‐risk adenomas. Moreover, the findings highlight POFUT1 and Notch signaling as potential drivers of colorectal tumor development.

**Abbreviations**
CAE: cancer‐associated event
CRC: colorectal cancer
EIF6: eukaryotic translation initiation factor 6
GSEA: gene set enrichment analysis
HRA: high‐risk adenoma
IHC: immunohistochemistry
LC–MS/MS: liquid chromatography tandem mass spectrometry
LRA: low‐risk adenoma
MSI: microsatellite instability/instable
MSS: microsatellite‐stable
POFUT1: protein O‐fucosyltransferase 1
RNA‐seq: RNA sequencing
RPRD1B: regulation of nuclear pre‐mRNA domain containing 1B
TMA: tissue microarray
WGS: whole genome sequencing

## Introduction

Colorectal adenomas are benign precursor lesions of colorectal cancer (CRC) that arise from normal epithelium.^1^ The prevalence of adenomas in the large intestine is much higher than the incidence of cancer,^2^, ^3^ implying that the majority of adenomas will never progress to CRC.^4^ In clinical practice, adenomas detected during colonoscopy are completely removed, and consequently the natural history of disease is disrupted. Based on the prevalence of focal cancer in endoscopically removed adenomas, it is estimated that only 5% of adenomas will eventually progress to CRC.^5^, ^6^ Currently, adenomas larger than 1 cm and/or with a villous component and/or with high‐grade dysplasia are referred to as “advanced adenomas” and are considered to be clinically relevant precursors of CRC. However, incidence studies of both advanced adenomas and CRCs suggest that these features alone are not precise predictors of the malignant progression.^7^

Cancer is caused by molecular alterations in DNA, thereby affecting gene expression at RNA and protein level. The “advanced adenoma” definition neglects molecular changes that accompany adenoma‐to‐carcinoma progression. In multiple cancer types, the progression of dysplastic epithelial premalignant lesions, like colorectal adenomas, has been associated with acquisition of genomic instability.^8^, ^9^ This often concerns chromosomal instability, which affects about 85% of CRCs.^10^ Studies on chromosomal instability in colorectal adenomas and cancers led to identification of nonrandom chromosomal aberrations and potential CRC driver events, which play a major role in adenoma‐to‐carcinoma progression.^11^, ^12^, ^13^, ^14^, ^15^, ^16^, ^17^, ^18^ Seven chromosomal copy number aberrations have been identified as colorectal cancer‐associated events (CAEs); gains of chromosomal arms 8q, 13q and 20q and losses of chromosomal arms 8p, 15q, 17p and 18q. With the accuracy of 78%, the presence of at least two of these CAEs enabled distinction of an adenoma with a focus of cancer from a nonmalignant adenoma.^11^ Therefore, adenomas with at least two out of the seven CAEs are marked as high risk of progressing to malignancy, further referred to as high‐risk adenomas (HRAs).^11^ We recently observed that only 23–36% of advanced adenomas classify as HRAs based on their DNA copy number profile.^19^

The aim of the present study was to characterize adenomas at low and high risk of progressing to cancer by molecular profiling at DNA, RNA and protein level, allowing to examine the biological processes in which these adenomas differ and to discover putative drivers of early colorectal tumor development.

## Materials and Methods

### Tissue data

Fresh frozen tissue material from 30 CRCs, 30 adenomas and 18 normal colorectal mucosa samples was collected at the Department of Pathology of the Amsterdam University Medical Center (VUmc) in Amsterdam, as described previously.^20^ Collection, storage and use of tissue and patient data were performed in compliance with the “Code for Proper Secondary Use of Human Tissue in the Netherlands” (https://www.federa.org/). All normal samples were adjacent to colorectal neoplasia; four normal colon samples were adjacent to adenomas and cancers, six to colorectal adenomas and eight to CRC. All normal samples were obtained from the furthest point from colorectal neoplasia within the surgically resected material and judged as 100% normal by an expert pathologist. In our study all adenomas were larger than 1 cm in size to allow sampling of fresh frozen material for research purposes from tissues that were collected for routine diagnostics. Therefore, all of the adenomas used in our study were “advanced adenomas.” For each sample, one tissue piece was cut into serial sections that were alternatingly used for DNA, RNA and protein isolation in the order DNA–RNA‐protein‐(…)‐DNA–RNA‐protein, to obtain the most comparable molecular profiles on DNA, RNA and protein level.

### Genomics data

Low‐coverage whole genome sequencing (WGS) data for the adenomas and RNA sequencing (RNA‐seq) data for colorectal adenomas and cancers were obtained in our previous study.^20^ For the normal adjacent colon sample collection, DNA and RNA isolation, low‐coverage WGS and RNA‐seq was performed as previously described for adenomas and cancers.^20^ Raw sequencing data were made available through the European Genome‐Phenome Archive (https://ega-archive.org/, EGAS00001002854). DNA copy number aberration identification in CRCs and normal adjacent colon samples was performed as described previously for the adenomas.^20^

### Mass spectrometry proteomics data

Sample preparation for liquid chromatography tandem mass spectrometry proteomics (LC–MS/MS) was performed as previously described,^21^ with some modifications (Supplementary Materials and Methods). Mass spectrometry was performed on a Q Exactive‐HF mass spectrometer (Thermo Fisher, Bremen, Germany) using a data independent acquisition mass spectrometry protocol. The data independent acquisition mass spectrometry method consisted of a MS1 scan from 400 to 1,000 m/z at 15,000 resolution (AGC target of 3 × 10^6^ and 50 ms injection time). For MS2, 24 variable size DIA segments were acquired at 30,000 resolution (AGC target 3 × 10^6^ and auto for injection time). The data independent acquisition mass spectrometry method included 20 windows of 20 m/z, 2 × 40 m/z and 2 × 60 m/z. Collision energy was set at 28%. The spectra were recorded in centroid mode. The default charge state for the MS2 was set to 3.

### RNA‐seq data analysis

RNA‐seq data preprocessing was performed as described previously,^20^ now using human genome build hg19 (USCS RefSeq hg19, gencode v19 annotation). RNA‐seq data were subjected to differential expression analysis, cellular decomposition (ESTIMATE^22^ algorithm), gene set enrichment analysis (GSEA)^23^ and gene‐dosage effect analysis (Supplementary Materials and Methods).

### Proteomics data analysis

An in‐house spectral library was established using LC–MS/MS data derived from CRCs, colorectal adenomas and normal adjacent colon samples (manuscript in preparation), which was used in Spectronaut^24^ to identify mass spectra. Protein groups were identified, quality control was performed and protein expression data was subjected to differential expression analysis, GSEA^23^ and gene‐dosage effect analysis (Supplementary Materials and Methods).

### Quantification of tumor‐stroma and goblet cells

Fresh‐frozen tissue sections taken “before” and “after” the tissue sections used for DNA, RNA and protein isolation were stained with hematoxylin and eosin, and scanned using Aperio AT2 Scanner (Leica Biosystems Imaging, Amsterdam, The Netherlands). The digital images were used for stroma and goblet cells quantification (Supplementary Materials and Methods).

### Immunohistochemical staining of tissue microarrays and patient‐derived colorectal adenoma organoids

Candidate drivers of adenoma‐to‐carcinoma progression were selected for immunohistochemical (IHC) validation of protein expression in colorectal tissues using tissue microarrays (TMAs), and in cultures of epithelial cells using sections of patient‐derived colorectal adenoma organoids. Candidates were selected using the following criteria: higher expression in HRAs when compared to low‐risk adenomas (LRAs); and higher intensity in CRCs when compared to normal colon according to the Human Protein Atlas (http://www.proteinatlas.org).^25^ See Supplementary Materials and Methods for details on IHC and patient‐derived organoids.

## Results

### Molecular characterization of LRA and HRA

**Figure 1:**
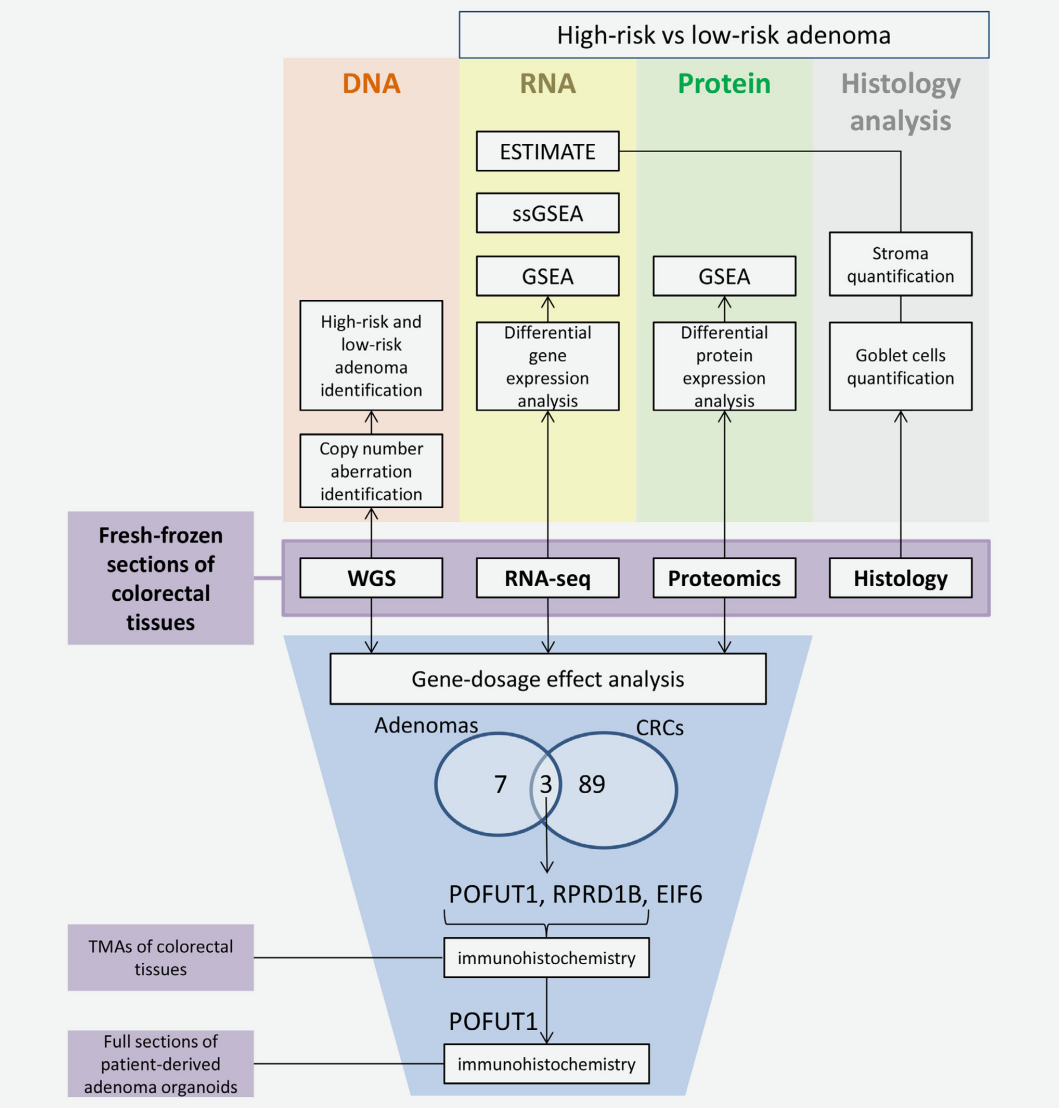
Fresh‐frozen tissue fragments of colorectal cancers (*n* = 30), colorectal adenomas (*n* = 30) and normal adjacent colon samples (*n* = 18) were used for low‐coverage WGS, RNA‐seq, tandem mass spectrometry proteomics and histology analysis. DNA copy number aberration identification and HRA and LRA stratification was performed using the low‐coverage WGS data. RNA‐seq and proteomics data were used for differential gene/protein expression analysis and GSEA. Additionally, single sample GSEA and ESTIMATE algorithm, which calculate the enrichment of stromal and immune gene signatures, were used on the RNA expression data set. Stroma quantification was performed on sections originating from the same tissue fragments as used for the molecular profiling data to validate the results of the expression analysis. Stroma percentage was compared between HRA and LRA and correlated with the stromal score of the ESTIMATE algorithm. Next, DNA copy number driven gene‐dosage effect analysis was performed. Ninety‐two and ten genes were identified to correlate in terms of DNA copy number, RNA and protein expression in CRCs and adenomas, respectively. Three genes, *POFUT1*, *RPRD1B* and *EIF6*, were overlapping between adenomas and cancers and were observed to be amplified and overexpressed in HRAs and CRCs. Validation of *POFUT1* and *RPRD1B* by immunohistochemical staining was performed in TMAs of the formalin‐fixed, paraffin‐embedded tissue pieces and for *POFUT1* also in full sections of patient‐derived adenoma organoids. Additionally, goblet cell quantification was performed on the sections of colorectal adenomas and association with *POFUT1* expression and risk of progression was identified. [Color figure can be viewed at http://wileyonlinelibrary.com]

With the aim to characterize colorectal adenomas in the context of colorectal tumor progression, we have performed low‐coverage WGS, genome‐wide RNA‐seq and tandem mass spectrometry proteomics (LC–MS/MS) on 30 colorectal adenomas,^20^ 30 CRCs and 18 adjacent normal colon tissues (see Fig. 1 for an overview of the analyses applied in the entire study and Supplementary Table S2 for clinical information on the samples). Using low‐coverage WGS we determined DNA copy number aberrations in the samples. Within the adenomas, nine HRAs were identified based on the presence of at least two CAEs. To obtain a robust representation of LRAs, only microsatellite‐stable (MSS) lesions that carried none of the CAEs were included. Two adenomas were microsatellite‐instable (MSI), two adenomas carried only one CAE, and for two adenomas the calling of CAEs remained inconclusive,^20^ leaving 15 MSS adenomas with no CAEs that were classified as LRAs (Supplementary Fig. S1*a* and Table S3). No significant associations were observed for risk of progression and pathological adenoma features like size, grade of dysplasia or histology (Table S4). CRCs showed the well‐known nonrandom pattern of chromosomal instability with CAEs being the most frequent, next to gain of chromosome 7 and loss of chromosome 14 (Fig. S1*a*). As six CRCs had previously been identified as MSI,^20^ the DNA copy number frequency for MSI CRCs and MSS CRCs were examined separately, revealing less chromosomal aberrations in MSI CRCs (Fig. S1*b*). No chromosomal aberrations were observed in the normal adjacent colon samples (Fig. S1*a*).

To explore the biological processes playing a role in colorectal tumor progression, the tissue samples were analyzed by RNA‐seq and LC–MS/MS. Mass spectrometry analysis lead to identification of 5,080 protein groups in the whole data set and 4,903 in the group of HRAs and LRAs (false discovery rate ≤0.01). Among the adenomas, one HRA was identified as an outlier due to low protein group number and highly differing expression profile from the rest of the adenoma samples (Fig. S2) and was excluded from further proteomic analyses. Dimensionality reduction of the RNA and protein expression data allowed to clearly discern adenomas from CRCs and normal adjacent colon tissues (Figs. S3*a* and S3*c*) while HRAs and LRAs were indistinguishable (Figs. S3*b* and S3*d*).

**Figure 2:**
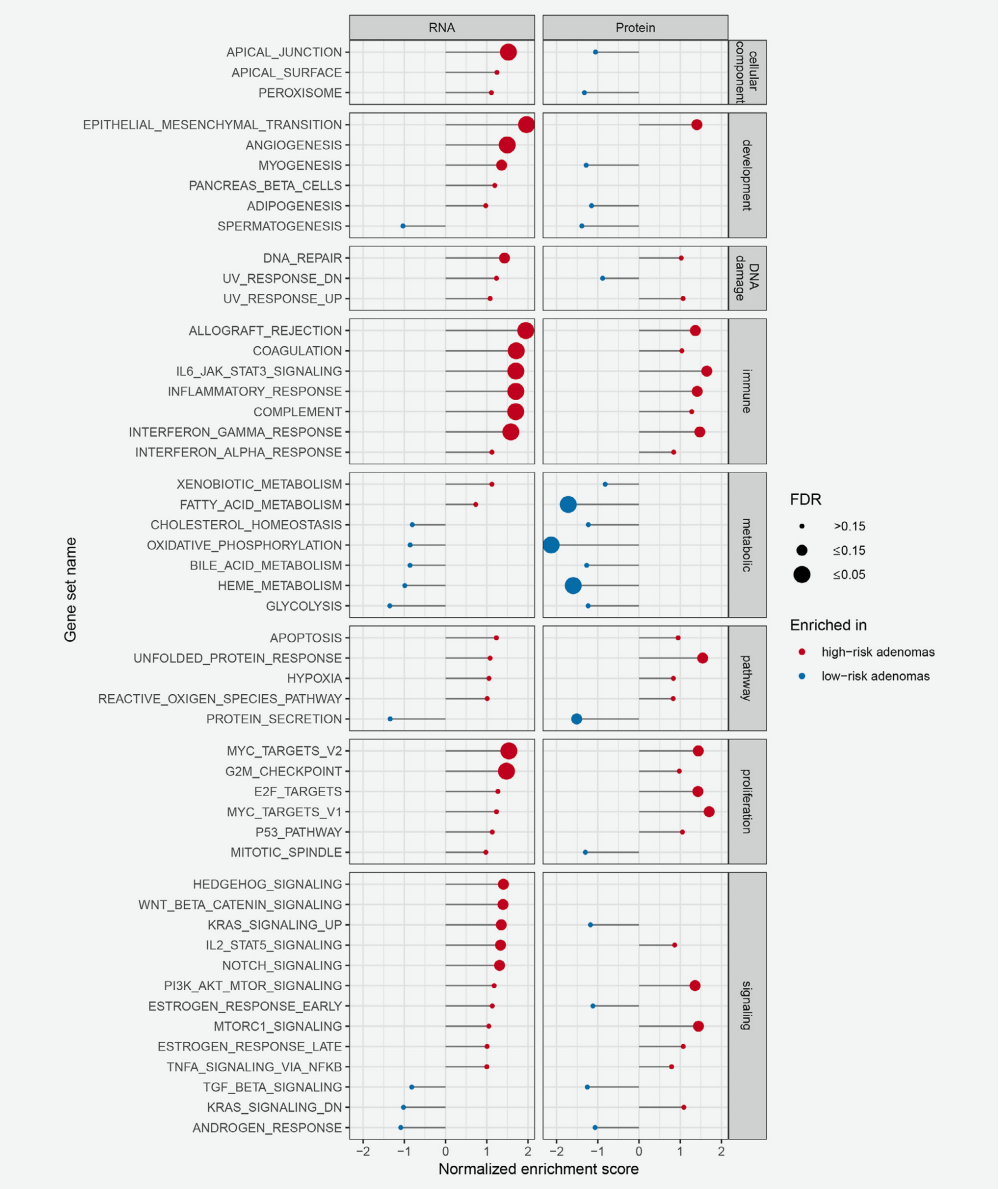
Gene set enrichment analysis results in the differential analysis between HRA and LRA, on RNA and protein level, as measured by RNA‐seq and mass spectrometry proteomics. Genes or proteins were ranked based on their fold change and *p*‐value, with genes/proteins significantly overexpressed in HRAs on top of the list. GSEA was performed on the ranked list using hallmark gene sets. Gene sets enriched in HRAs are marked red, and gene sets enriched in LRAs are marked blue. The size of the dot reflects the significance of the enrichment (false discovery rate ≤0.15). For a subset of the signaling pathways, like Hedgehog, Wnt and Notch, GSEA on the protein level could not be determined since the number of proteins from these gene sets identified by LC–MS/MS was too small. [Color figure can be viewed at http://wileyonlinelibrary.com]

Differential gene expression analysis between the HRAs and LRAs revealed 298 genes with higher and 125 genes with lower expression in HRAs (Table S5). Differential protein expression analysis revealed 78 proteins with higher and 86 with lower expression in HRAs (Table S6). Fourteen genes were differentially expressed on both RNA and protein level, with 9 genes higher and 5 lower expressed in HRAs (Table S7). To gain further insights into the global differences between the adenomas, we performed GSEA with hallmark gene signatures (molecular signature database^26^) on lists of genes and proteins ranked according to differences in the expression between HRAs and LRAs (Fig. 2). Processes that were more prominent in HRAs on RNA and protein level were related to proliferation, immune response and stroma development. Additionally, a number of signaling pathways were enriched in HRAs either only on the RNA (KRAS‐signaling up, Hedgehog‐, WNT‐, IL2‐STAT5‐, NOTCH‐signaling’ or protein level (PI3K/AKT/mTOR‐, mTORC1‐signaling). The processes more prominent in LRAs compared to HRAs were identified on the protein level and included “protein secretion” and the metabolic gene sets (Fig. 2).

**Figure 3:**
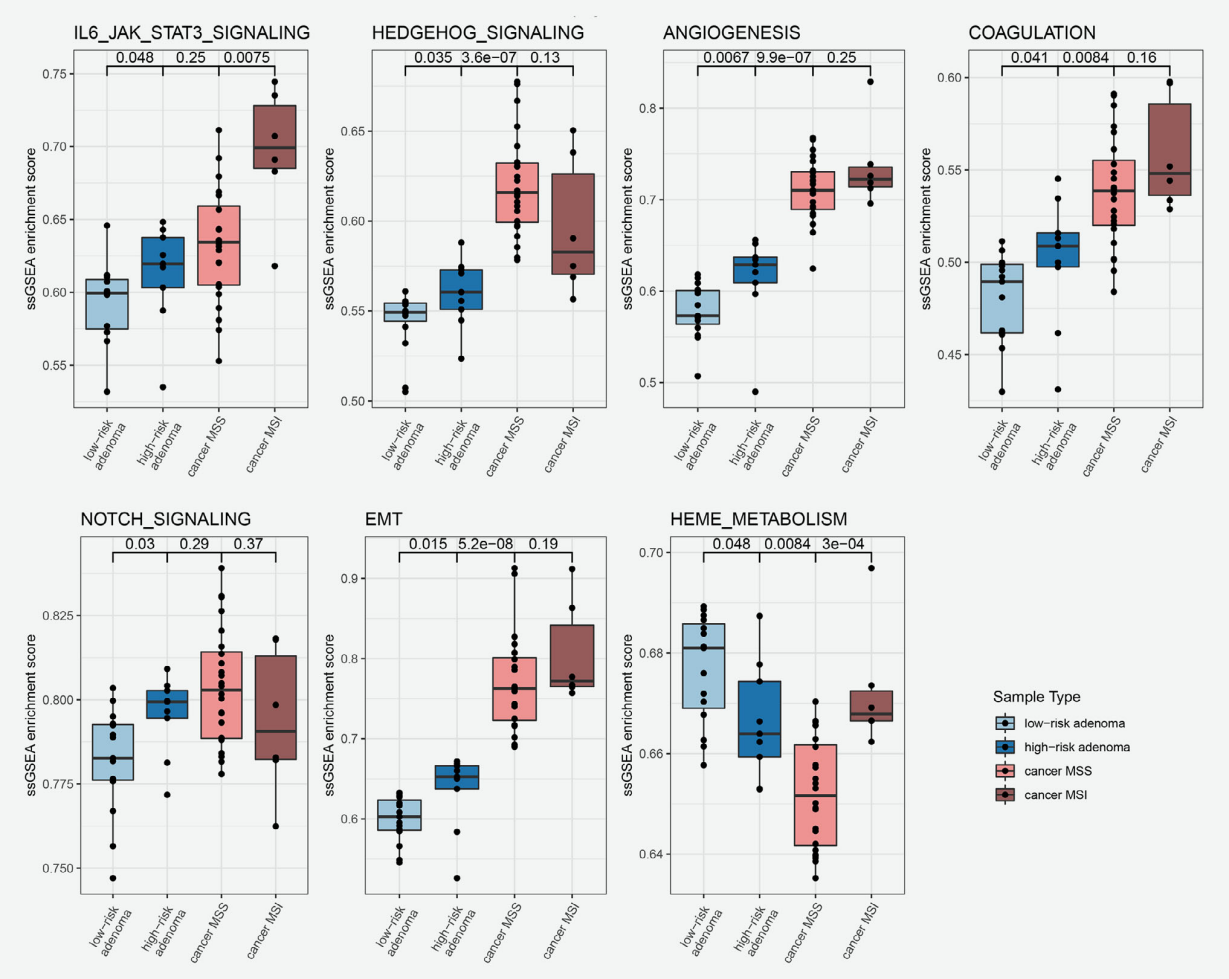
Single sample gene set enrichment scores represented per sample type; LRAs, HRAs and CRCs. Gene sets with significant differences in enrichment scores between HRA and LRA (*p* ≤ 0.05) were selected for this figure. [Color figure can be viewed at http://wileyonlinelibrary.com]

To put the GSEA group‐level differences between HRAs and LRAs in context of progression toward CRC, we performed single‐sample GSEA on RNA level in adenomas and cancers using the hallmark gene sets (Fig. S4). Seven gene sets were significantly differential between HRAs and LRAs (*p* ≤ 0.05, Fig. 3). In six cases, the single‐sample GSEA score increased through colorectal tumor progression, with the lowest score in LRAs and the highest in CRCs. These include “Notch‐” and “Hedgehog‐signaling” together with immune‐ and stroma‐related gene sets, like “epithelial‐mesenchymal transition.” For “heme metabolism,” the single‐sample GSEA score decreased through colorectal tumor progression (Fig. 3).

### Characterization of LRA and HRA tumor microenvironment

As GSEA revealed increased stroma and immune processes in HRAs, we examined the differences in tumor microenvironment between HRAs and LRAs. By applying the ESTIMATE algorithm^22^ on RNA expression data, enrichment scores for stromal and immune signatures were calculated in each sample reflecting the expression of stroma‐ and immune‐related genes (Fig. S5). A significant increase of stromal score was identified in HRAs when compared to LRAs (*p* = 0.012). An even more significant increase was observed between MSS cancers and HRAs (*p* = 5.7e^−5^). In terms of the immune score, even though a gradual increase from LRAs through HRAs to MSS cancers was identified, the differences between the groups were insignificant (*p* = 0.096 and 0.98, respectively). MSI cancers had significantly higher immune score than MSS cancer (*p* = 0.021, Fig. S5).

**Figure 4:**
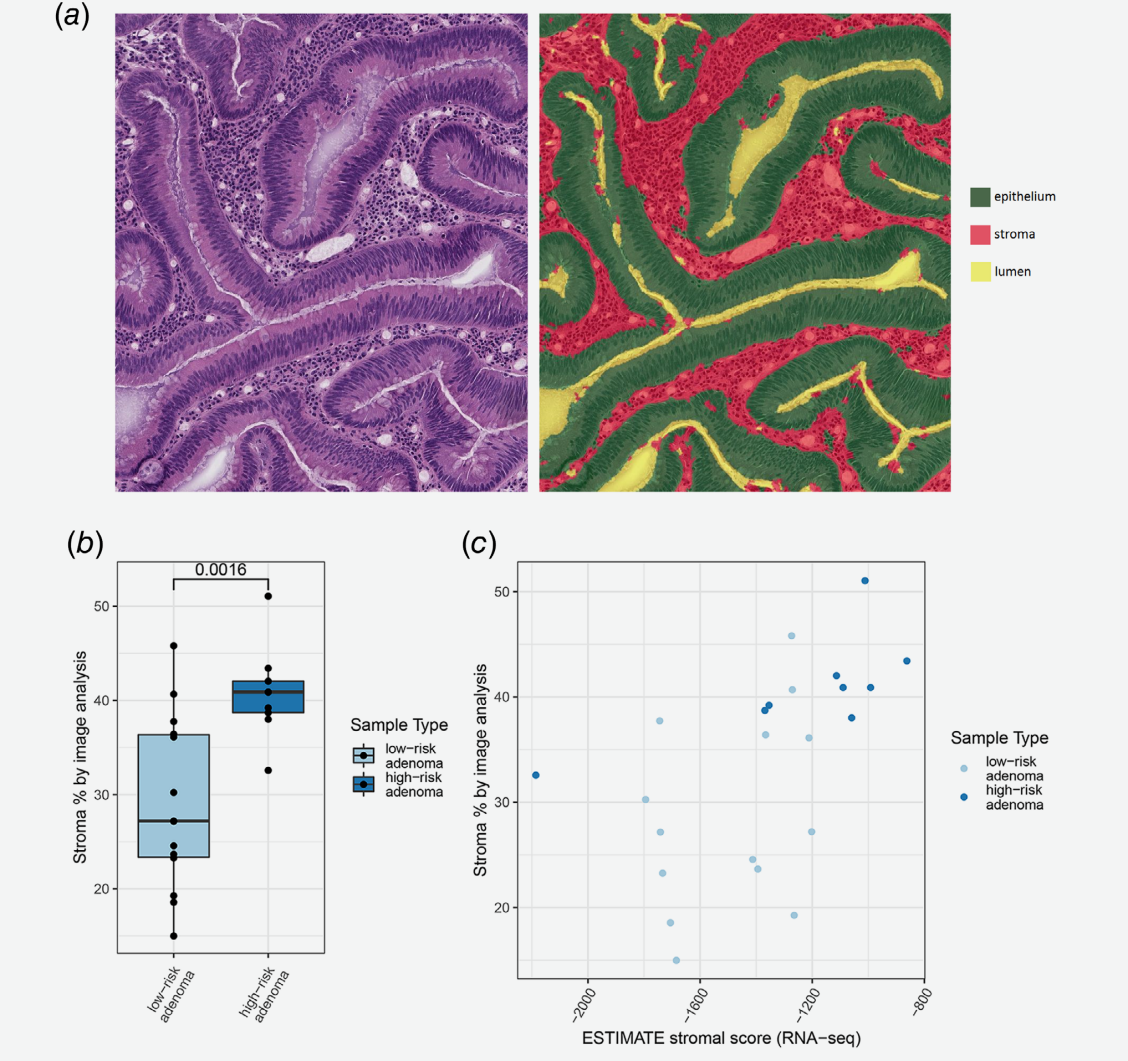
Stroma quantification on hematoxylin and eosin‐stained slides. (*a*) Representative image of assigning class to area on the slide; stroma, epithelium or lumen. Each class was quantified by calculating the size of its area. (*b*) Significant difference in stroma percentage between HRA and LRA, as calculated by the image analysis. (*c*) Significant positive correlation identified between stroma percentage measured by image analysis and ESTIMATE stromal score. [Color figure can be viewed at http://wileyonlinelibrary.com]

To morphologically confirm the differences in the amounts of stroma between the HRAs and LRAs, we performed stroma quantification on hematoxylin and eosin‐stained slides by digital image analysis (Fig. 4 *a*). One sample could not be analyzed due to excessive tissue folds. The amount of stroma in HRAs (median = 40.89) was significantly higher than in LRAs (median = 27.20, *p* = 0.002, Fig. 4 *b*). Stroma percentage calculated by image analysis also positively correlated with the ESTIMATE stromal score from the RNA expression analysis (Fig. 4 *c*). This indicates that the expression differences between HRAs and LRAs in stromal and immune pathways are associated with the morphological differences in the amount of stroma in the tissue samples.

### Candidate drivers of adenoma‐to‐carcinoma progression

Next to identification of differences in tumor microenvironment, we investigated DNA copy number driven gene‐dosage effect to reveal changes between HRAs and LRAs driven by the aberrations in the epithelial cells (Fig. 1). Pairwise correlation analysis was performed between DNA copy number, RNA and protein expression for colorectal adenomas and CRCs. In the cancers, 92 genes were positively correlated among the data types (Fig. S6 and Table S8). Chromosome 20 was associated with the largest global expression changes on RNA and protein level with 28 genes (~30%), including *HNF4A*, *TOMM34* and *RPRD1B*, which were previously described to be gained and overexpressed in CRC cell lines and tissues.^27^, ^28^ Gene‐dosage effect was also identified for *DIS3*, which is located on chromosome 13 and often gained in CRC.^27^, ^29^ Other genomic regions with the highest number of perturbed genes considered almost all chromosomes involved in the CAEs.

**Figure 5:**
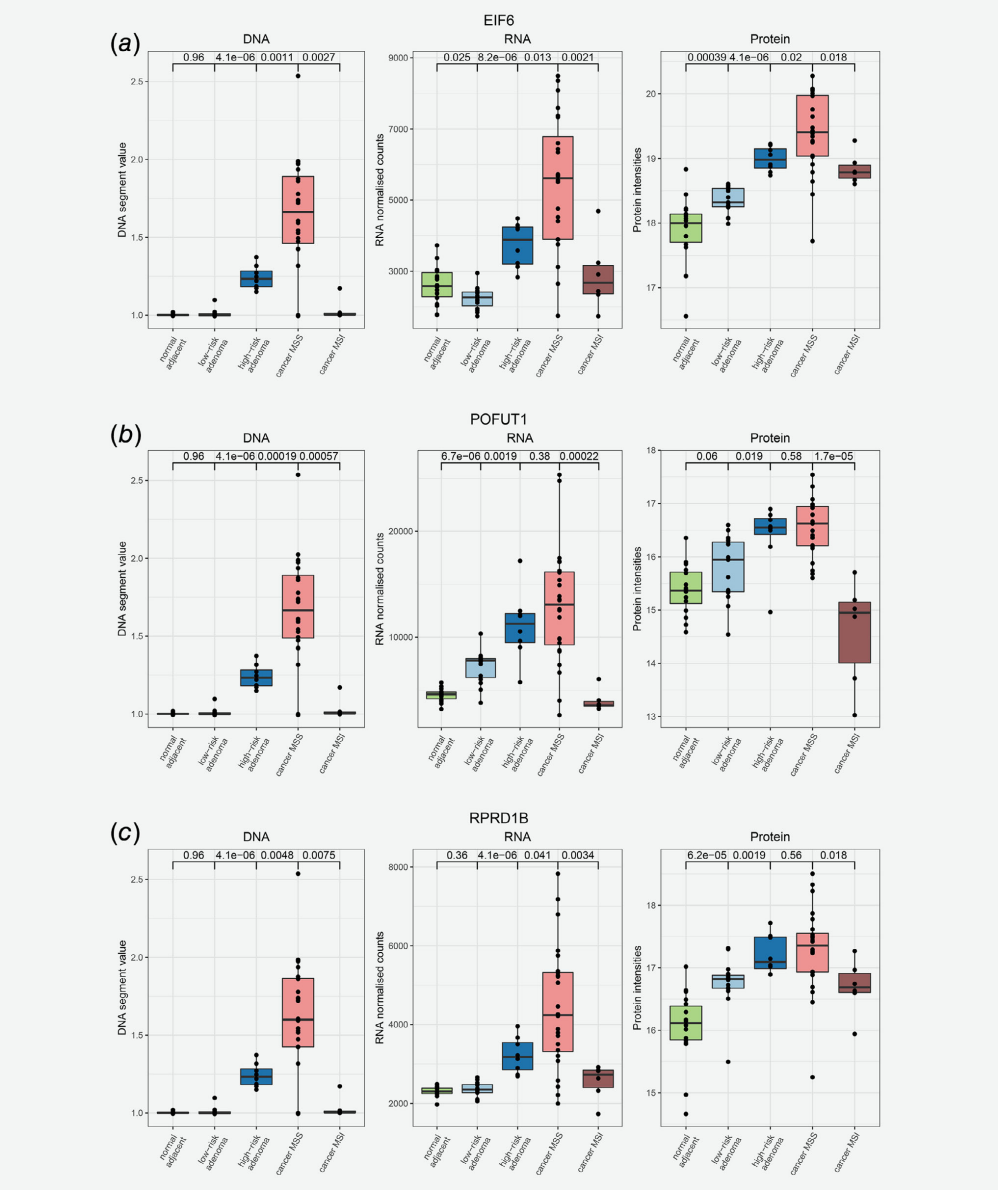
Proteogenomic representation of the potential drivers of colorectal tumors. DNA copy number, RNA and Protein expression (as measured by mass spectrometry proteomics) were plotted for EIF6 (*a*), POFUT1 (*b*) and RPRD1B (*c*) for each sample among different stages of colorectal tumor development: normal adjacent colon, LRAs, HRAs and CRCs. Correlating, gradual increase in DNA copy number and RNA and Protein expression was observed for each of these three genes. [Color figure can be viewed at http://wileyonlinelibrary.com]

In the adenomas, positive and significant correlations between DNA copy numbers, RNA and protein expression were identified for 10 genes (Fig. S6 and Table S9). As HRAs are characterized by presence of CAEs, potential drivers of early colorectal tumor progression are expected to reside on the CAE‐defined chromosomes. Gene‐dosage effect was identified for two genes from chromosome arm 8p; however, these genes were associated both with gains and losses in the HRA group (Fig. S1*a*) and consequently, higher and lower gene and protein expression when compared to LRAs. For the genes located on the CAE‐related chromosome 20, *POFUT1*, *RPRD1B* and *EIF6*, gene‐dosage effect was associated with only gains (Fig. S1*a*) and overexpression in HRAs when compared to LRAs (Fig. 5). We performed gene‐level overlap analysis between gene‐dosage effects in CRCs and in adenomas to identify genes prominent for both HRAs and CRCs. The analysis revealed *POFUT1*, *RPRD1B* and *EIF6*, implying that the gain of chromosome arm 20q and expression of these three genes play an important role in both HRAs and CRCs. For all of these three genes DNA copy number, RNA and protein expression increased gradually from normal adjacent colon, through LRAs and HRAs to CRCs (Fig. 5). *POFUT1*, *RPRD1B* and *EIF6* reside on neighboring cytogenetic bands—20q11.21, 20q11.23 and 20q11.22, respectively. Moreover, significant positive correlations were identified between these genes on DNA, RNA and protein level, suggesting their coamplification and coexpression (Fig. S7).

To validate gene‐dosage effect of *POFUT1*, *EIF6* and *RPRD1B* in colorectal tumors, we evaluated the relation between DNA copy numbers, RNA and protein expression of these genes in The Cancer Genome Atlas (TCGA) Provisional CRC data set.^30^, ^31^ Gene‐dosage effect was confirmed for each of these three genes in this data set on both RNA (*n* = 382) and protein level (*n* = 90), as gene and protein expression was higher when the DNA copy of the gene was gained or amplified (Figs. S8–S10).

### Validation of increased POFUT1 expression in HRAs

To verify whether protein expression of *POFUT1*, *RPRD1B* and *EIF6* is increased in CRCs and HRAs compared to LRAs and normal colon tissue, we aimed to evaluate their expression by immunohistochemistry (IHC) using TMAs obtained from the same samples as were used for the molecular profiling. Data in the Human Protein Atlas^25^ indicated that the expression of EIF6, as measured by IHC, is already high in normal colon tissue, leaving little room to detect increased EIF6 protein expression in adenomas and CRCs. Therefore, TMAs were stained for POFUT1 and RPRD1B, while EIF6 was discarded from IHC analysis.

Within the TMA cores of colorectal tissues, RPRD1B was observed mainly in the nuclei of epithelial cells (Fig. S8), the staining confirmed increasing protein expression of RPRD1B in HRAs and CRCs as observed in the molecular profiling data (Fig. 5 *c*). Nevertheless, several LRAs and normal adjacent colon samples exhibited high intensity of RPRD1B staining (Fig. S11 and Table S10). Therefore, the difference in RPRD1B expression measured by IHC between LRAs and HRAs was not significant (*p* = 0.197; Table S10). Comparisons of CRCs with HRAs to LRAs and of CRCs with HRAs to LRAs with normal colon samples yielded significant differences (*p* = 0.017 and 0.003, respectively; Table S10).

**Figure 6:**
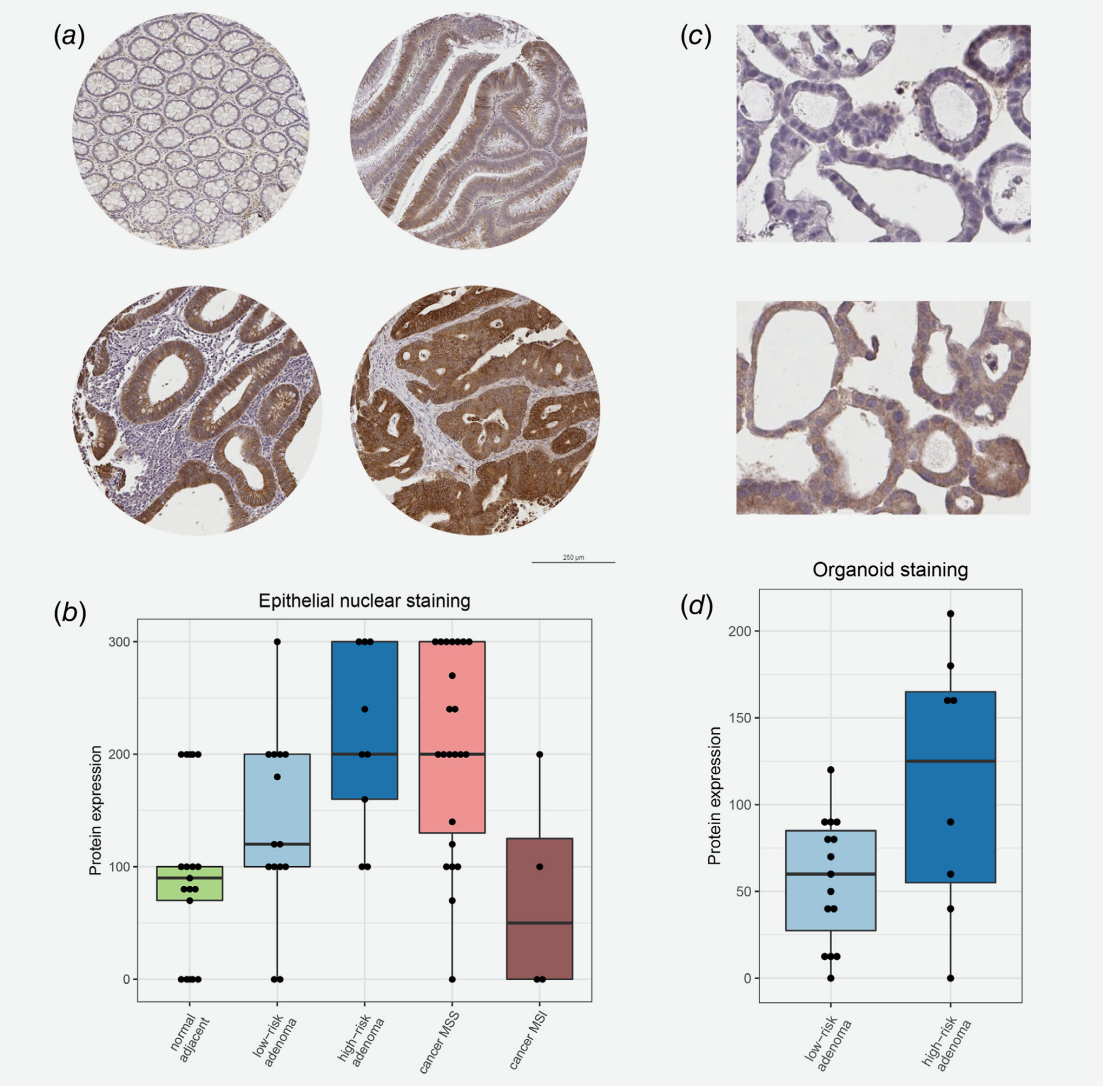
Immunohistochemical staining of POFUT1 in colorectal tissues and patient‐derived organoids. (*a*) Representative POFUT1 staining in different tissue sample type. Top left: normal adjacent colon; top right: LRA; bottom left: HRA; bottom right: CRC. (*b*) POFUT1 expression as measured by a product of epithelial cytoplasmic staining intensity (negative = 0, weak = 1, moderate = 2 or strong = 3) and percentage of the cells stained positively (0–100%) was plotted for each tissue sample among different stages of colorectal tumor development. See Table S11 for group comparisons and statistical testing. (*c*) Representative images of POFUT1 staining in LRA organoid (top) and HRA organoid (bottom). (*d*) POFUT1 expression in epithelial cytoplasm plotted in HRA and LRA organoids, as measured by a product of epithelial cytoplasmic staining intensity (negative = 0, weak = 1, moderate = 2 or strong = 3) and percentage of the cells stained positively (0–100%). See Table S13 for group comparisons and statistical testing. [Color figure can be viewed at http://wileyonlinelibrary.com]

POFUT1 immunohistochemical staining was predominantly observed in the cytoplasm of epithelial cells, the staining showed gradual increase of POFUT1 expression through different stages of colorectal tumor progression (Figs. 6 *a* and 6 *b*), thereby verifying the molecular profiling data (Fig. 5 *b*). High levels of POFUT1 expression measured by IHC were more frequent in HRAs compared to LRAs, in HRAs and cancers compared to LRAs and in HRAs and cancers compared to LRAs and normal adjacent colon (Tables S11*a* and S11*b*). POFUT1 expression was also significantly associated with grade of dysplasia (Table S11*b*). Interestingly, POFUT1 expression was lower in MSI than in MSS cancers on both RNA and protein level (Figs. 5 *b* and 6), suggesting its specific role for chromosomal instability tumors. Previously, depletion of *POFUT1* was shown to play a role in differentiation of the proliferative epithelial cells into goblet cells through inactivation of Notch signaling.^32^ Therefore, we quantified the amount of goblet cells in the adenomas using hematoxylin and eosin‐stained sections to examine this finding in the context of risk of progression. No association of the amount of goblet cells with dysplasia or other pathological features was identified (Table S11*b*). Lower amounts of goblet cells were significantly associated with high POFUT1 expression (*p* = 0.017; Table S11*a*) and high risk of progression (*p* = 0.007; Table S11*b*), implying that also in our study *POFUT1* is linked to goblet cell differentiation and indicating its role in early colorectal tumor development.

To further corroborate the role of *POFUT1* in the pathogenesis of CRC in an independent series, expression of POFUT1 was investigated in a cohort of patient‐derived colorectal adenoma organoids. First, we performed low‐coverage WGS and based on the presence of two or more CAEs revealed 8 HRA and 15 LRA organoids in the series (Table S12). Next, IHC staining of the organoids was performed to evaluate POFUT1 expression in the neoplastic cells. Also in the organoids, POFUT1 was mainly observed in the cytoplasm and high POFUT1 expression was associated with HRAs (*p* = 0.008; Table S13 and Figs. 6 *c* and 6 *d*), confirming its potential role in early colorectal tumor development.

## Discussion

Studying the natural history of colorectal adenomas, including progression to cancer, is challenging because adenomas are removed when detected during colonoscopy. Yet, there is a need for better understanding of the biology of adenomas that progress to CRC. We set out to molecularly characterize adenomas at high risk of progressing to CRC and to identify putative drivers of this process. *POFUT1* was found to be amplified and overexpressed in HRAs and CRCs when compared to LRAs and adjacent normal colon epithelium. POFUT1 overexpression was successfully validated by immunohistochemical staining on TMAs and in patient‐derived colorectal adenoma organoids, indicating that POFUT1 plays a role in colorectal adenoma‐to‐carcinoma progression. Additionally, high POFUT1 expression and high risk of progression to cancer were associated with a decrease in goblet cell differentiation.

The novelty of the current study is multi‐omics analysis of colorectal adenomas at high and low risk of progressing to cancer, in the context of CRCs and normal adjacent colon samples. Comprehensive analysis of high throughput DNA, RNA and protein profiling data of the same samples has not been performed yet for colorectal adenomas, while it did provide additional insights in CRC.^27^, ^28^ On RNA and/or protein level, the enrichment of gene sets and pathways were identified to be increasing through different stages of colorectal tumor development, from normal colon, through LRA and HRA to CRC. These included pathways known to play a role in or accompany colorectal carcinogenesis like Hedgehog, Notch, KRAS, PI3K/AKT/mTOR or Wnt signaling, proliferation, epithelial‐mesenchymal transition or immune activation.^33^ This suggests that a lot of processes inherent to cancer are already more active in HRAs compared to LRAs. Conversely, gene sets enriched in LRAs when compared to HRAs, like protein secretion, fatty‐acid or heme metabolism, decreased in CRC, consistent with previous observations.^34^ Fourteen genes were identified to be differentially expressed between HRAs and LRAs on both RNA and protein level. Among upregulated genes/proteins in HRAs, genes of both epithelial and stromal origins were found. This included *HNF4A*, a transcriptional activator of epithelial differentiation^35^ that is located on chromosomal arm 20q, previously shown to be amplified and activated in the majority of CRCs^28^ and studied as a prognostic biomarker for this disease.^36^ An unexpected result was the overexpression of multiple tumor microenvironment‐related genes/proteins in HRAs, including collagens, fibronectin, vimentin, immunoglobulins or calprotectin. While a broad range of stroma proportion has been reported in CRC,^37^ this is far less evident in adenomas. It has been shown that stromal genes can be expressed by epithelial cells, which typically occurs in association with invasion, a phenomenon referred to as epithelial‐mesenchymal transition.^35^ Nevertheless, by definition, stroma invasion is a process characteristic to cancer and not yet occurring in adenomas. We have performed stroma quantification by image analysis on adenoma tissue sections originating from the same tissue fragments that were used for molecular profiling, and observed a significant increase in stroma percentage in HRAs compared LRAs. Our data indicate that differential expression of the stroma genes between HRAs and LRAs is likely due to the differences in the stroma proportion. Even though significant, the variation in the amount of stroma in the adenomas is certainly not as big as in CRCs.^37^

To identify putative drivers of adenoma‐to‐carcinoma progression from the epithelial cells, we examined DNA‐driven aberrations in the colorectal tumors. Combining DNA and RNA data to study gene‐dosage effect has been performed in CRC^18^; however, only for a limited number of potential candidates functional assays confirmed their oncogenic potential.^14^, ^29^, ^38^ Addition of the protein layer provides insight into which chromosomal aberrations lead to functional consequences.^28^ Despite the high depth of the proteomics measurement in the present study with over 5,000 protein groups detected in total, adding the protein layer can be also limiting, in terms of the number of proteins measured overall and subsequently considered in the analysis. In our study, gene‐dosage effect analysis in CRCs led to the identification of 92 genes, a subset of which has previously been described, including *HNF4A*,^28^ *TOMM34*,^28^ *DIS3* 29 or *RPRD1B*.^27^

In the adenomas, the CAE‐driven gene‐dosage effect analysis yielded potential drivers of colorectal tumor progression that are already amplified and overexpressed in HRAs—*POFUT1*, *RPRD1B* and *EIF6*. The three genes are located on neighboring cytobands of chromosome arm 20q, which is the most frequently amplified chromosomal arm in CRC.^18^, ^28^

POFUT1 is a fucosylation factor that activates Notch through addition of fucose groups,^39^ a process required for the canonical Notch signaling.^32^, ^40^ In our study, *POFUT1* was amplified and overexpressed while Notch signaling was enriched in HRAs and CRCs, when compared to LRAs. High expression of POFUT1 in HRAs and CRCs was validated using immunohistochemical staining of TMAs and adenoma‐derived organoids. Recently, *POFUT1* overexpression was shown to have oncogenic activity in CRC through activation of *NOTCH1* signaling, and consequently affecting proliferation, invasion and migration.^41^ Additionally, depletion of *POFUT1* or Notch signaling was shown to be associated with converting proliferative cells into goblet cells.^32^, ^42^ Indeed, in the present study, low numbers of goblet cells were significantly associated with high‐risk status and high POFUT1 expression in adenomas, indicating that in HRAs *POFUT1* and Notch signaling play a role in increased proliferation and decreased differentiation. Altogether this suggests that *POFUT1* through the Notch signaling pathway is a putative driver of adenoma‐to‐carcinoma progression. Further functional studies on adenoma preclinical models are needed to confirm this hypothesis.

*RPRD1B* is overexpressed in many tumor types and has been shown to have an oncogenic activity by regulating the transcription of cyclin D1^43^ and other Wnt targets,^44^ consistent with the significant enrichment of Wnt signaling in HRAs demonstrated by GSEA in the present study. *RPRD1B* was proven to accelerate tumorigenesis by promoting cell proliferation and invasion.^43^, ^44^ Altogether, this suggests that *RPRD1B* may play a role in colorectal tumor progression through enhanced Wnt signaling. Although the TMA IHC analyses did not validate differences in RPRD1B expression levels between LRA and HRA, its predominant staining of neoplastic cells combined with the molecular profiling data suggest that *RPRD1B* should also be considered as a putative driver of colorectal tumor development.

EIF6 is a translation initiation factor that plays a role in ribosome complex formation and protein synthesis downstream of PI3K/Akt/mTOR signaling pathway.^45^, ^46^ It is overexpressed in multiple tumor types,^47^, ^48^ including CRC, where expression of EIF6 has been shown to increase from normal colon, through adenoma to CRC.^49^ Functional studies on *EIF6* suggest its oncogenic activity through increasing cancer cell motility and invasion.^50^, ^51^ The fact that we identified significant enrichment of PI3K/Akt/mTOR signaling in HRAs when compared to LRAs, suggests that *EIF6* and PI3K/Akt/mTOR signaling play a role in adenoma‐to‐carcinoma progression. Additionally, the transcription of *EIF6* has been shown to be regulated by *NOTCH1*,^51^ consistent with Notch signaling enrichment in HRAs and CRCs.

Individuals with a history of colorectal neoplasia carry an increased risk of developing CRC in the future and therefore are enrolled in the colonoscopy‐based surveillance programs.^52^ As removal of nonmalignant precursor lesions during colonoscopy is an approach to decrease CRC incidence and mortality rates,^53^ currently, detection of advanced adenoma is an indication to shorten the interval for the follow‐up surveillance colonoscopy.^52^ The high prevalence of advanced adenomas in an elderly population leads to a substantial burden on endoscopic capacity.^52^ Moreover, given that not all advanced adenomas eventually progress to cancer, frequent surveillance colonoscopies in patients with these lesions lead to overdiagnosis and overtreatment.^4^ In our study, we have shown that HRAs, in contrast to LRAs, in a number of aspects resemble CRCs on molecular level, while they represent only approximately 30% of the advanced adenomas.^19^ Introduction of a more specific definition of adenomas associated with risk of future CRC development may significantly improve the CRC surveillance programs and reduce patient burden. Additional studies are still needed to evaluate if patients with HRAs indeed have higher CRC incidence and mortality rate compared to patients with advanced adenomas, and whether POFUT1 can be used as biomarker to identify HRAs in the surveillance setting.

In our study, we performed multi‐omics characterization of colorectal adenomas in the context of colorectal tumor development. We focused on conventional chromosomal instability adenomas, the most prevalent precursors of CRC,^10^ as MSI adenomas are relatively rare with a prevalence of only 3%.^54^ MSI CRCs were included in our analyses, which frequently differed from MSS CRCs in terms of gene expression and GSEA, confirming the distinct etiology of MSS and MSI CRCs. *POFUT1*, *RPRD1B* and *EIF6* were identified as putative drivers of adenoma‐to‐carcinoma progression. In light of what is known about the roles these genes play in carcinogenesis, our results imply that the transition from LRAs to HRAs involves the interplay of Wnt, Notch and PI3K/AKT/mTOR signaling pathways. As such, our study shows that biological processes inherent to CRC are already more active in HRAs than in LRAs. Moreover, our study emphasizes the key role that specific DNA copy number alterations play in progression from premalignancy to cancer, indicating that in comparison to the generally used morphology‐based concept of “advanced adenoma,” the molecular CAE‐based concept of HRA is a more specific marker to define risk of progressing to CRC.

## Supporting information


**Appendix S1**: Supplementary Tables

**Appendix S2**: Supplementary Figures

**Appendix S3**: Supplementary Material

## Data Availability

Raw sequencing data were made available through the European Genome‐Phenome Archive (https://ega-archive.org/, EGAS00001002854). The mass spectrometry proteomics data have been deposited to the ProteomeXchange Consortium *via* the PRIDE partner repository with the accession identifier PXD012254.

## NGS‐ProToCol consortium members

Natasja Dits, Department of Urology, Erasmus Medical Center Rotterdam, Rotterdam, The Netherlands.

René Böttcher, Department of Urology, Erasmus Medical Center Rotterdam, Rotterdam, The Netherlands.

Annemieke C. Hiemstra, Department of Pathology, Netherlands Cancer Institute, Amsterdam, The Netherlands.

Bauke Ylstra, Department of Pathology, Amsterdam UMC, Vrije Universiteit Amsterdam, Amsterdam, The Netherlands.

Daoud Sie, Department of Pathology, Amsterdam UMC, Vrije Universiteit Amsterdam, Amsterdam, The Netherlands.

Evert van den Broek, Department of Pathology, Netherlands Cancer Institute, Amsterdam, The Netherlands.

Nicole van Grieken, Department of Pathology, Amsterdam UMC, Vrije Universiteit Amsterdam, Amsterdam, The Netherlands.

David van der Meer, GenomeScan, Leiden, The Netherlands.

Floor Pepers, GenomeScan, Leiden, The Netherlands.

Eric Caldenhoven, Lygature, Utrecht, The Netherlands.

Bart Janssen, GenomeScan, Leiden, The Netherlands.

Wilbert van Workum, GenomeScan, Leiden, The Netherlands.

Stef van Lieshout, Department of Pathology, Amsterdam UMC, Vrije Universiteit Amsterdam, Amsterdam, The Netherlands.

Chris H. Bangma, Department of Urology, Erasmus Medical Center Rotterdam, Rotterdam, The Netherlands.

Geert van Leenders, Department of Pathology, Erasmus Medical Center Rotterdam, Rotterdam, The Netherlands.

Harmen J.G. van de Werken, Department of Urology, Erasmus Medical Center Rotterdam, Rotterdam, The Netherlands; and Computational Biology Center, Erasmus Medical Center Rotterdam, Rotterdam, The Netherlands.

## References

[1] Fearon ER , Vogelstein B . A genetic model for colorectal tumorigenesis. Cell 1990;61:759–67. 2188735 10.1016/0092-8674(90)90186-i

[2] Lieberman DA , Weiss DG , Bond JH , et al. Use of colonoscopy to screen asymptomatic adults for colorectal cancer. Veterans Affairs Cooperative Study Group 380. N Engl J Med 2000;343:162–8. 10900274 10.1056/NEJM200007203430301

[3] Imperiale TF , Wagner DR , Lin CY , et al. Risk of advanced proximal neoplasms in asymptomatic adults according to the distal colorectal findings. N Engl J Med 2000;343:169–74. 10900275 10.1056/NEJM200007203430302

[4] Kalager M , Wieszczy P , Lansdorp‐Vogelaar I , et al. Overdiagnosis in colorectal cancer screening: time to acknowledge a blind spot. Gastroenterology 2018;155:592–5. 30076834 10.1053/j.gastro.2018.07.037

[5] Muto T , Bussey HJ , Morson BC . The evolution of cancer of the colon and rectum. Cancer 1975;36:2251–70. 1203876 10.1002/cncr.2820360944

[6] Shinya H , Wolff WI . Morphology, anatomic distribution and cancer potential of colonic polyps. Ann Surg 1979;190:679–83. 518167 10.1097/00000658-197912000-00001 PMC1345622

[7] Brenner H , Hoffmeister M , Stegmaier C , et al. Risk of progression of advanced adenomas to colorectal cancer by age and sex: estimates based on 840 149 screening colonoscopies. Gut 2007;56:1585–9. 17591622 10.1136/gut.2007.122739 PMC2095643

[8] Heselmeyer K , Schrock E , du Manoir S , et al. Gain of chromosome 3q defines the transition from severe dysplasia to invasive carcinoma of the uterine cervix. Proc Natl Acad Sci U S A 1996;93:479–84. 8552665 10.1073/pnas.93.1.479 PMC40262

[9] Ried T , Just KE , Holtgreve‐Grez H , et al. Comparative genomic hybridization of formalin‐fixed, paraffin‐embedded breast tumors reveals different patterns of chromosomal gains and losses in fibroadenomas and diploid and aneuploid carcinomas. Cancer Res 1995;55:5415–23. 7585611

[10] Rajagopalan H , Nowak MA , Vogelstein B , et al. The significance of unstable chromosomes in colorectal cancer. Nat Rev Cancer 2003;3:695–701. 12951588 10.1038/nrc1165

[11] Hermsen M , Postma C , Baak J , et al. Colorectal adenoma to carcinoma progression follows multiple pathways of chromosomal instability. Gastroenterology 2002;123:1109–19. 12360473 10.1053/gast.2002.36051

[12] Meijer GA , Hermsen MA , Baak JP , et al. Progression from colorectal adenoma to carcinoma is associated with non‐random chromosomal gains as detected by comparative genomic hybridisation. J Clin Pathol 1998;51:901–9. 10070331 10.1136/jcp.51.12.901 PMC501025

[13] Douglas EJ , Fiegler H , Rowan A , et al. Array comparative genomic hybridization analysis of colorectal cancer cell lines and primary carcinomas. Cancer Res 2004;64:4817–25. 15256451 10.1158/0008-5472.CAN-04-0328

[14] Sillars‐Hardebol AH , Carvalho B , Tijssen M , et al. TPX2 and AURKA promote 20q amplicon‐driven colorectal adenoma to carcinoma progression. Gut 2012;61:1568–75. 22207630 10.1136/gutjnl-2011-301153

[15] Camps J , Grade M , Nguyen QT , et al. Chromosomal breakpoints in primary colon cancer cluster at sites of structural variants in the genome. Cancer Res 2008;68:1284–95. 18316590 10.1158/0008-5472.CAN-07-2864 PMC4729303

[16] Hirsch D , Camps J , Varma S , et al. A new whole genome amplification method for studying clonal evolution patterns in malignant colorectal polyps. Genes Chromosomes Cancer 2012;51:490–500. 22334367 10.1002/gcc.21937 PMC3535186

[17] Ried T , Knutzen R , Steinbeck R , et al. Comparative genomic hybridization reveals a specific pattern of chromosomal gains and losses during the genesis of colorectal tumors. Genes Chromosomes Cancer 1996;15:234–45. 8703849 10.1002/(SICI)1098-2264(199604)15:4<234::AID-GCC5>3.0.CO;2-2

[18] Carvalho B , Postma C , Mongera S , et al. Multiple putative oncogenes at the chromosome 20q amplicon contribute to colorectal adenoma to carcinoma progression. Gut 2009;58:79–89. 18829976 10.1136/gut.2007.143065

[19] Carvalho B , Diosdado B , Terhaar Sive Droste JS , et al. Evaluation of cancer‐associated DNA copy number events in colorectal (advanced) adenomas. Cancer Prev Res 2018;11:403–12. 10.1158/1940-6207.CAPR-17-0317 29685877

[20] Komor MA , Bosch LJ , Bounova G , et al. Consensus molecular subtypes classification of colorectal adenomas. J Pathol 2018;246:266–76. 29968252 10.1002/path.5129 PMC6221003

[21] Bosch LJW , de Wit M , Pham TV , et al. Novel stool‐based protein biomarkers for improved colorectal cancer screening: a case‐control study. Ann Intern Med 2017;167:855–66. 29159365 10.7326/M17-1068

[22] Yoshihara K , Shahmoradgoli M , Martínez E , et al. Inferring tumour purity and stromal and immune cell admixture from expression data. Nat Commun 2013;4:2612. 24113773 10.1038/ncomms3612 PMC3826632

[23] Subramanian A , Tamayo P , Mootha VK , et al. Gene set enrichment analysis: a knowledge‐based approach for interpreting genome‐wide expression profiles. Proc Natl Acad Sci U S A 2005;102:15545–50. 16199517 10.1073/pnas.0506580102 PMC1239896

[24] Bruderer R , Bernhardt OM , Gandhi T , et al. Extending the limits of quantitative proteome profiling with data‐independent acquisition and application to acetaminophen treated 3D liver microtissues. Mol Cell Proteomics 2015;14:1400–10. 25724911 10.1074/mcp.M114.044305 PMC4424408

[25] Uhlen M , Zhang C , Lee S , et al. A pathology atlas of the human cancer transcriptome. Science 2017;357:eaan2507. 28818916 10.1126/science.aan2507

[26] Liberzon A , Birger C , Thorvaldsdóttir H , et al. The molecular signatures database hallmark gene set collection. Cell Syst 2015;1:417–25. 26771021 10.1016/j.cels.2015.12.004 PMC4707969

[27] Wang J , Mouradov D , Wang X , et al. Colorectal cancer cell line proteomes are representative of primary tumors and predict drug sensitivity. Gastroenterology 2017;153:1082–95. 28625833 10.1053/j.gastro.2017.06.008 PMC5623120

[28] Zhang B , Wang J , Wang X , et al. Proteogenomic characterization of human colon and rectal cancer. Nature 2014;513:382–7. 25043054 10.1038/nature13438 PMC4249766

[29] de Groen FL , Krijgsman O , Tijssen M , et al. Gene‐dosage dependent overexpression at the 13q amplicon identifies DIS3 as candidate oncogene in colorectal cancer progression. Genes Chromosomes Cancer 2014;53:339–48. 24478024 10.1002/gcc.22144

[30] Cerami E , Gao J , Dogrusoz U , et al. The cBio cancer genomics portal: an open platform for exploring multidimensional cancer genomics data. Cancer Discov 2012;2:401–4. 22588877 10.1158/2159-8290.CD-12-0095 PMC3956037

[31] Gao J , Aksoy BA , Dogrusoz U , et al. Integrative analysis of complex cancer genomics and clinical profiles using the cBioPortal. Sci Signal 2013;6:pl1. 23550210 10.1126/scisignal.2004088 PMC4160307

[32] Guilmeau S , Flandez M , Bancroft L , et al. Intestinal deletion of Pofut1 in the mouse inactivates Notch signaling and causes entero‐colitis. Gastroenterology 2008;135:849–60. e6. 18621050 10.1053/j.gastro.2008.05.050 PMC3207497

[33] Sillars‐Hardebol AH , Carvalho B , de Wit M , et al. Identification of key genes for carcinogenic pathways associated with colorectal adenoma‐to‐carcinoma progression. Tumour Biol 2010;31:89–96. 20358421 10.1007/s13277-009-0012-1 PMC2848338

[34] Carvalho B , Sillars‐Hardebol AH , Postma C , et al. Colorectal adenoma to carcinoma progression is accompanied by changes in gene expression associated with ageing, chromosomal instability, and fatty acid metabolism. Cell Oncol (Dordr) 2012;35:53–63. 22278361 10.1007/s13402-011-0065-1 PMC3308003

[35] Vellinga TT , den Uil S , Rinkes IH , et al. Collagen‐rich stroma in aggressive colon tumors induces mesenchymal gene expression and tumor cell invasion. Oncogene 2016;35:5263–71. 26996663 10.1038/onc.2016.60

[36] Chellappa K , Robertson GR , Sladek FM . HNF4α: a new biomarker in colon cancer? Biomark Med 2012;6:297–300. 22731903 10.2217/bmm.12.23 PMC3581081

[37] Fijneman RJ , Carvalho B , Postma C , et al. Loss of 1p36, gain of 8q24, and loss of 9q34 are associated with stroma percentage of colorectal cancer. Cancer Lett 2007;258:223–9. 17977645 10.1016/j.canlet.2007.09.013

[38] Camps J , Pitt JJ , Emons G , et al. Genetic amplification of the Notch modulator LNX2 upregulates the WNT/beta‐catenin pathway in colorectal cancer. Cancer Res 2013;73:2003–13. 23319804 10.1158/0008-5472.CAN-12-3159 PMC4729305

[39] Li Z , Han K , Pak JE , et al. Recognition of EGF‐like domains by the Notch‐modifying O‐fucosyltransferase POFUT1. Nat Chem Biol 2017;13:757–63. 28530709 10.1038/nchembio.2381

[40] Shi S , Stanley P . Protein O‐fucosyltransferase 1 is an essential component of Notch signaling pathways. Proc Natl Acad Sci U S A 2003;100:5234–9. 12697902 10.1073/pnas.0831126100 PMC154328

[41] Du Y , Li D , Li N , et al. POFUT1 promotes colorectal cancer development through the activation of Notch1 signaling. Cell Death Dis 2018;9:995. 30250219 10.1038/s41419-018-1055-2 PMC6155199

[42] van Es JH , van Gijn ME , Riccio O , et al. Notch/gamma‐secretase inhibition turns proliferative cells in intestinal crypts and adenomas into goblet cells. Nature 2005;435:959–63. 15959515 10.1038/nature03659

[43] Lu D , Wu Y , Wang Y , et al. CREPT accelerates tumorigenesis by regulating the transcription of cell‐cycle‐related genes. Cancer Cell 2012;21:92–104. 22264791 10.1016/j.ccr.2011.12.016

[44] Zhang Y , Liu C , Duan X , et al. CREPT/RPRD1B, a recently identified novel protein highly expressed in tumors, enhances the beta‐catenin.TCF4 transcriptional activity in response to Wnt signaling. J Biol Chem 2014;289:22589–99. 24982424 10.1074/jbc.M114.560979 PMC4132767

[45] Golob‐Schwarzl N , Schweiger C , Koller C , et al. Separation of low and high grade colon and rectum carcinoma by eukaryotic translation initiation factors 1, 5 and 6. Oncotarget 2017;8:101224–43. 29254159 10.18632/oncotarget.20642 PMC5731869

[46] Biffo S , Manfrini N , Ricciardi S . Crosstalks between translation and metabolism in cancer. Curr Opin Genet Dev 2018;48:75–81. 29153483 10.1016/j.gde.2017.10.011

[47] Rosso P , Cortesina G , Sanvito F , et al. Overexpression of p27BBP in head and neck carcinomas and their lymph node metastases. Head Neck 2004;26:408–17. 15122657 10.1002/hed.10401

[48] Miluzio A , Oliveto S , Pesce E , et al. Expression and activity of eIF6 trigger malignant pleural mesothelioma growth in vivo. Oncotarget 2015;6:37471–85. 26462016 10.18632/oncotarget.5462 PMC4741942

[49] Sanvito F , Vivoli F , Gambini S , et al. Expression of a highly conserved protein, p27BBP, during the progression of human colorectal cancer. Cancer Res 2000;60:510–6. 10676626

[50] Pinzaglia M , Montaldo C , Polinari D , et al. eIF6 over‐expression increases the motility and invasiveness of cancer cells by modulating the expression of a critical subset of membrane‐bound proteins. BMC Cancer 2015;15:131. 25886394 10.1186/s12885-015-1106-3 PMC4381359

[51] Benelli D , Cialfi S , Pinzaglia M , et al. The translation factor eIF6 is a Notch‐dependent regulator of cell migration and invasion. PLoS One 2012;7:e32047. 22348144 10.1371/journal.pone.0032047 PMC3279413

[52] Hassan C , Quintero E , Dumonceau JM , et al. Post‐polypectomy colonoscopy surveillance: European Society of Gastrointestinal Endoscopy (ESGE) guideline. Endoscopy 2013;45:842–51. 24030244 10.1055/s-0033-1344548

[53] Pan J , Xin L , Ma YF , et al. Colonoscopy reduces colorectal cancer incidence and mortality in patients with non‐malignant findings: a meta‐analysis. Am J Gastroenterol 2016;111:355–65. 26753884 10.1038/ajg.2015.418 PMC4820666

[54] Kinzler KW , Vogelstein B . Lessons from hereditary colorectal cancer. Cell 1996;87:159–70. 8861899 10.1016/s0092-8674(00)81333-1

