# Supplementary material for: Molecular characterization of colorectal adenomas reveals POFUT1 as a candidate driver of tumor progression
Source: Int J Cancer. 2019 Aug 30;146(7):1979–92. doi: 10.1002/ijc.32627 (PMC7027554; doi:10.1002/ijc.32627)

Supplementary Figures

Supplementary Figure 1. Frequency plots of DNA copy number aberrations. **A.** From top, normal adjacent colon (n=18), low-risk adenomas (n=15), high-risk adenomas (n=9) and cancers (n=30) **B.** MSI cancers (n=6) and MSS cancers (n=24).

Supplementary Figure 2. Quality assessment of proteomics data. **A**. Number of protein groups identified in each adenoma sample. Mean number of protein groups is highlighted with green solid line, mean minus 2 standard deviations of the number of protein groups is highlighted with green dashed line. The outlier sample (NGS-002-A) is the only one with the number of protein groups below the dashed line. **B.** Multidimensional scaling of adenoma samples based on the protein expression. The same outlier is highlighted.

Supplementary Figure 3. Multidimensional scaling of the RNA and protein expression profiles. **A**. Visualization of normal adjacent colon samples (green), adenomas (blue) and cancers (pink) based on the RNA expression profile. **B.** Visualization of high-risk (dark blue) and low-risk adenomas (light blue) based on the RNA expression profile. **C.** Visualization of normal adjacent colon samples, adenomas and cancers based on the protein expression profile. **D.** Visualization of high-risk and low-risk adenomas based on the protein expression profile.

Supplementary Figure 4. Single sample GSEA analysis in low-risk adenomas, high-risk adenomas and CRCs (MSS and MSI CRCs presented separately). All gene sets with insignificant difference between low-risk and high-risk adenomas are presented based on the Mann-Whitney test.

Supplementary Figure 5. Cellular decomposition based on RNA expression data. Stromal and immune enrichment scores as calculated by the ESTIMATE algorithm in low-risk and high-risk adenomas and cancers (MSS and MSI CRCs presented separately). P-values were obtained with the Mann-Whitney test.

Supplementary Figure 6. DNA copy number driven gene dosage effect in cancers (**A**) and adenomas (**B**). Pairwise correlation analysis was performed between DNA copy number, RNA and protein expression. Significantly correlating genes (FDR $\leq$ 0.1 or 0.25 for cancers and adenomas, respectively) on DNA, RNA and protein level were identified and grouped per chromosome they reside on. The number of correlating genes was plotted per chromosome.

Supplementary Figure 7. Pearson correlation analysis between DNA segment value, RNA normalized counts and normalized protein intensities of EIF6, POFUT1 and RPRD1B. The correlation analysis was performed on all the samples, including normal adjacent colon, adenoma and cancer samples. Bottom left matrix presents bivariate scatter plots with a fitted line. Top right displays correlation coefficient and significance level, where "***" means p-value ≤ 0.001.

Supplementary Figure 8. DNA copy number driven gene dosage effect in the TCGA colorectal adenomacarcinomas for POFUT1 identified on RNA (A) and protein (B) level. Each dot represents a sample and is grouped according to the DNA copy number of POFUT1. RNA (A) and protein (B) expression values in a form of Z-scores are plotted per group.

Supplementary Figure 9. DNA copy number driven gene dosage effect in the TCGA colorectal adenomacarcinomas for EIF6 identified on RNA (A) and protein (B) level. Each dot represents a sample and is grouped according to the DNA copy number of EIF6. RNA (A) and protein (B) expression values in a form of Z-scores are plotted per group.

Supplementary Figure 10. DNA copy number driven gene dosage effect in the TCGA colorectal adenomacarcinomas for RPRD1B identified on RNA (A) and protein (B) level. Each dot represents a sample and is grouped according to the DNA copy number of RPRD1B. RNA (A) and protein (B) expression values in a form of Z-scores are plotted per group.

Supplementary Figure 11. Immunohistochemical staining of RPRD1B in colorectal tissues. **A.** Representative RPRD1B staining in different types of tissue samples. Top left: normal adjacent colon, top right: low-risk adenoma, bottom left: high-risk adenoma, bottom right: colorectal cancer. **B.** RPRD1B expression as measured by a product of epithelial nuclear staining intensity (negative=0, weak=1, moderate=2 or strong=3) and percentage of the cells stained positively (0-100%) was plotted for the different types of tissue samples: normal adjacent colon, low-risk adenomas, high-risk adenomas and CRCs (MSS and MSI CRCs presented separately). See Supplementary Table 10 for group comparisons and statistical testing.

Supplementary Figure 1A

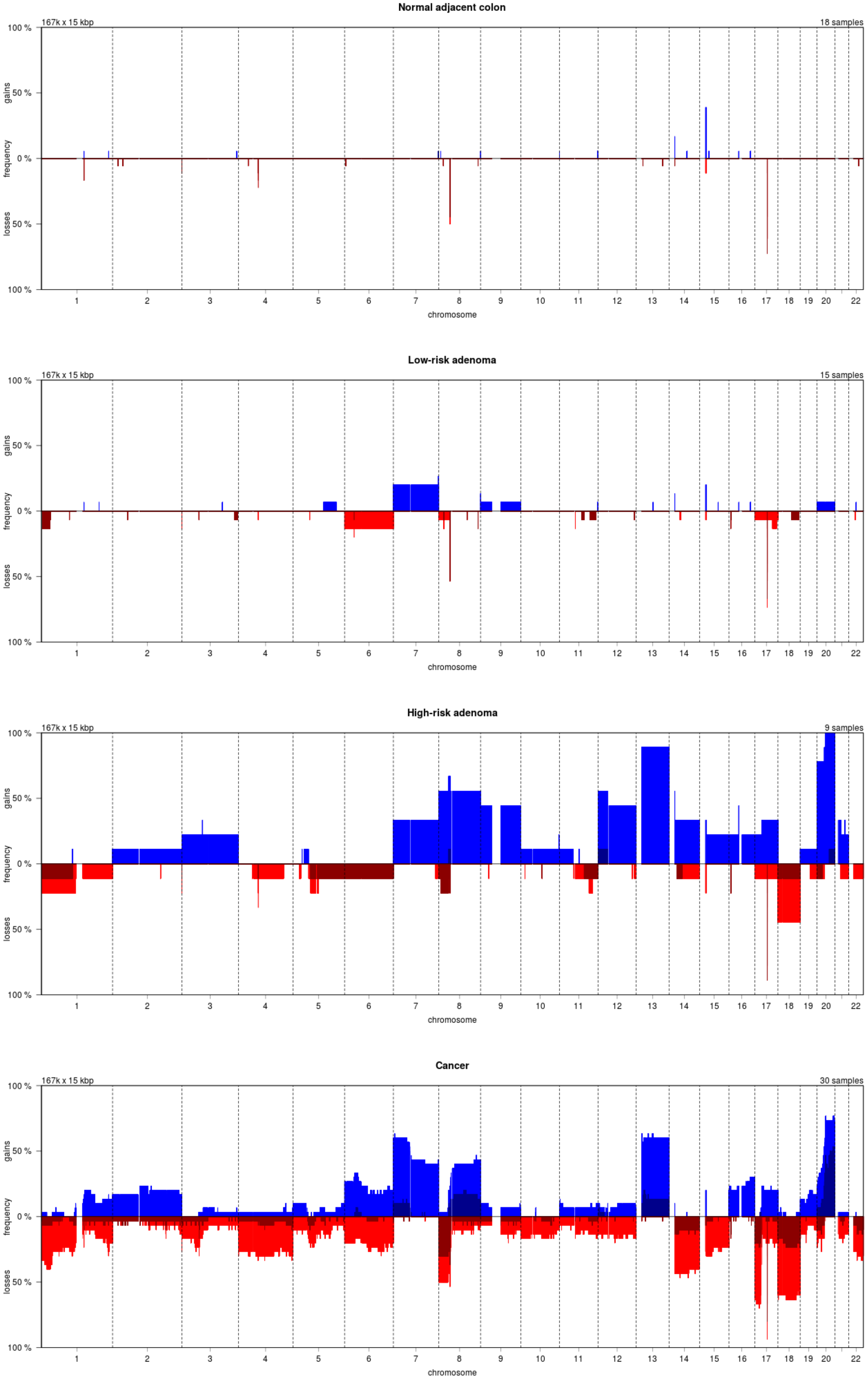

Supplementary Figure 1B

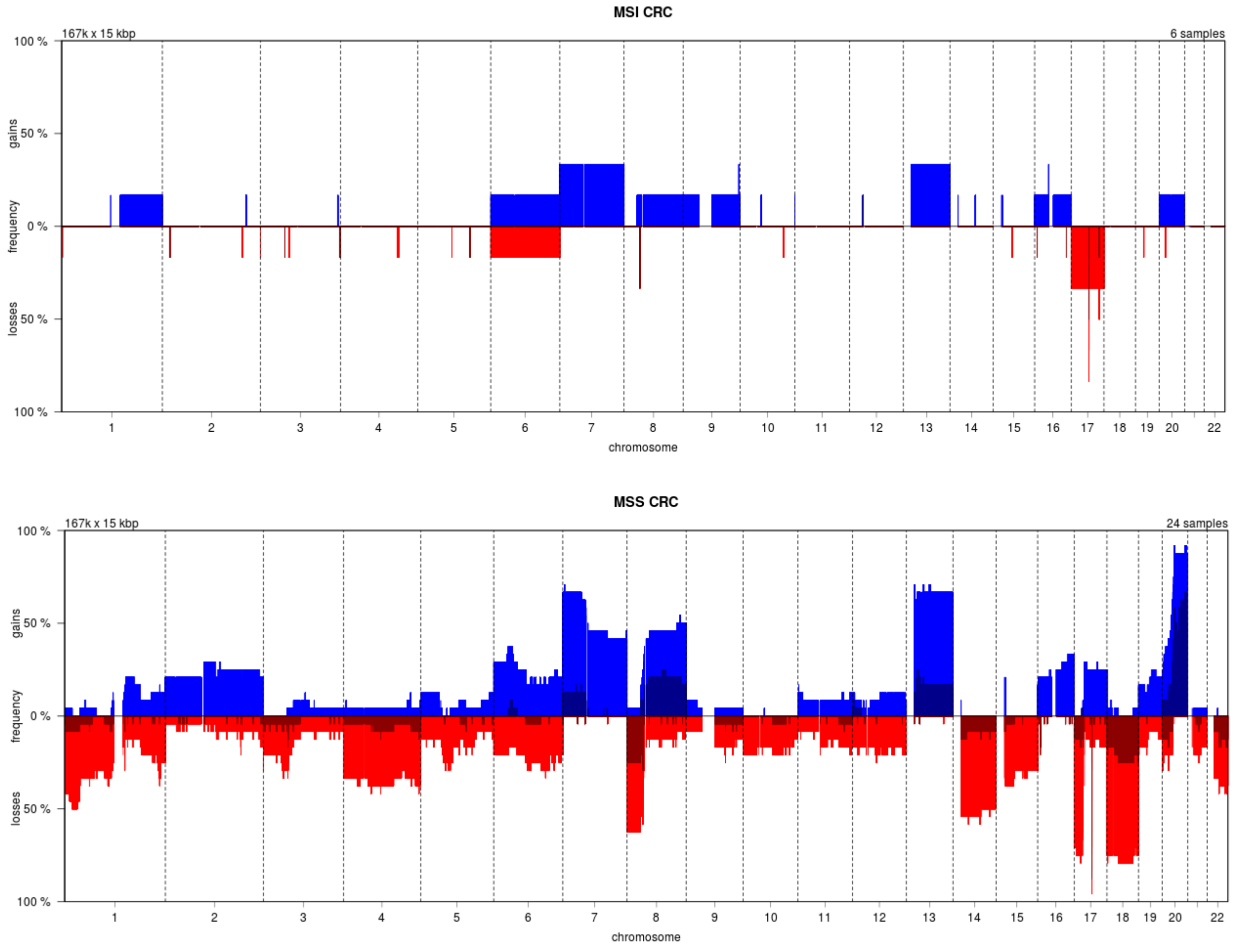

Supplementary Figure 2

A

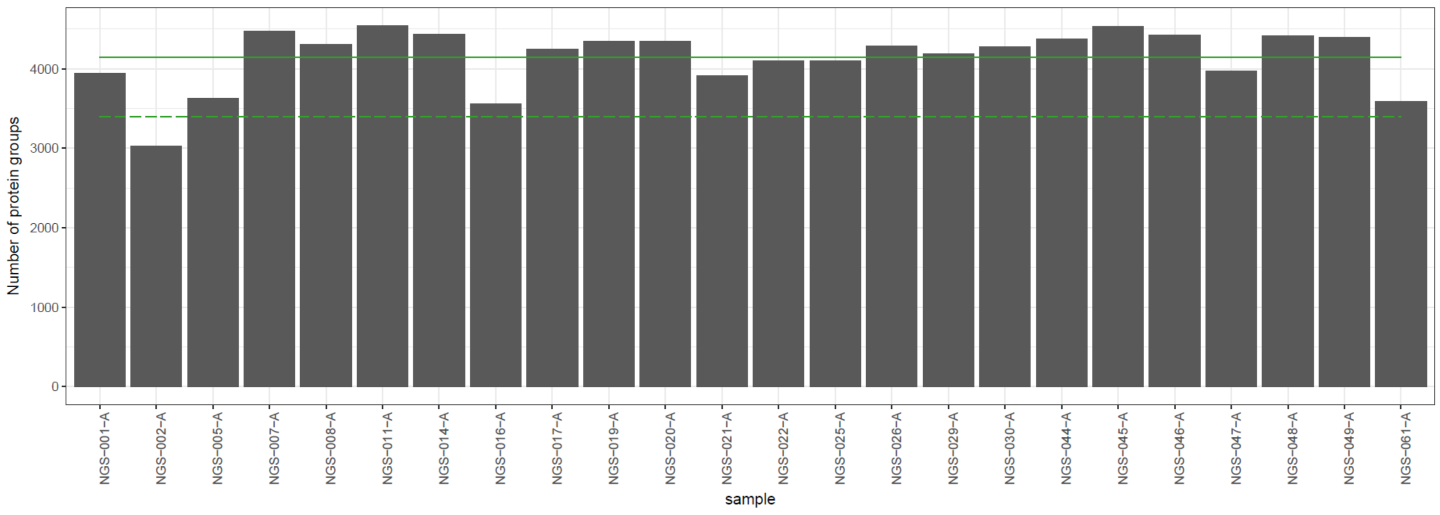

B

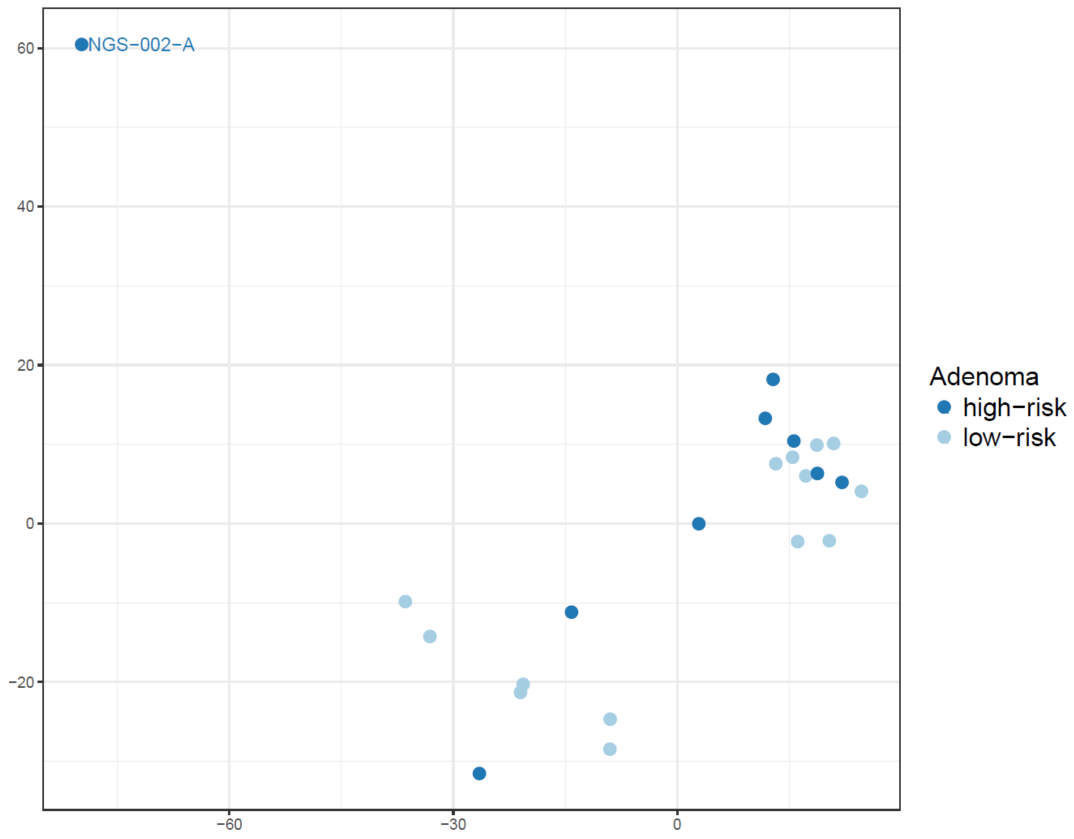

Supplementary Figure 3

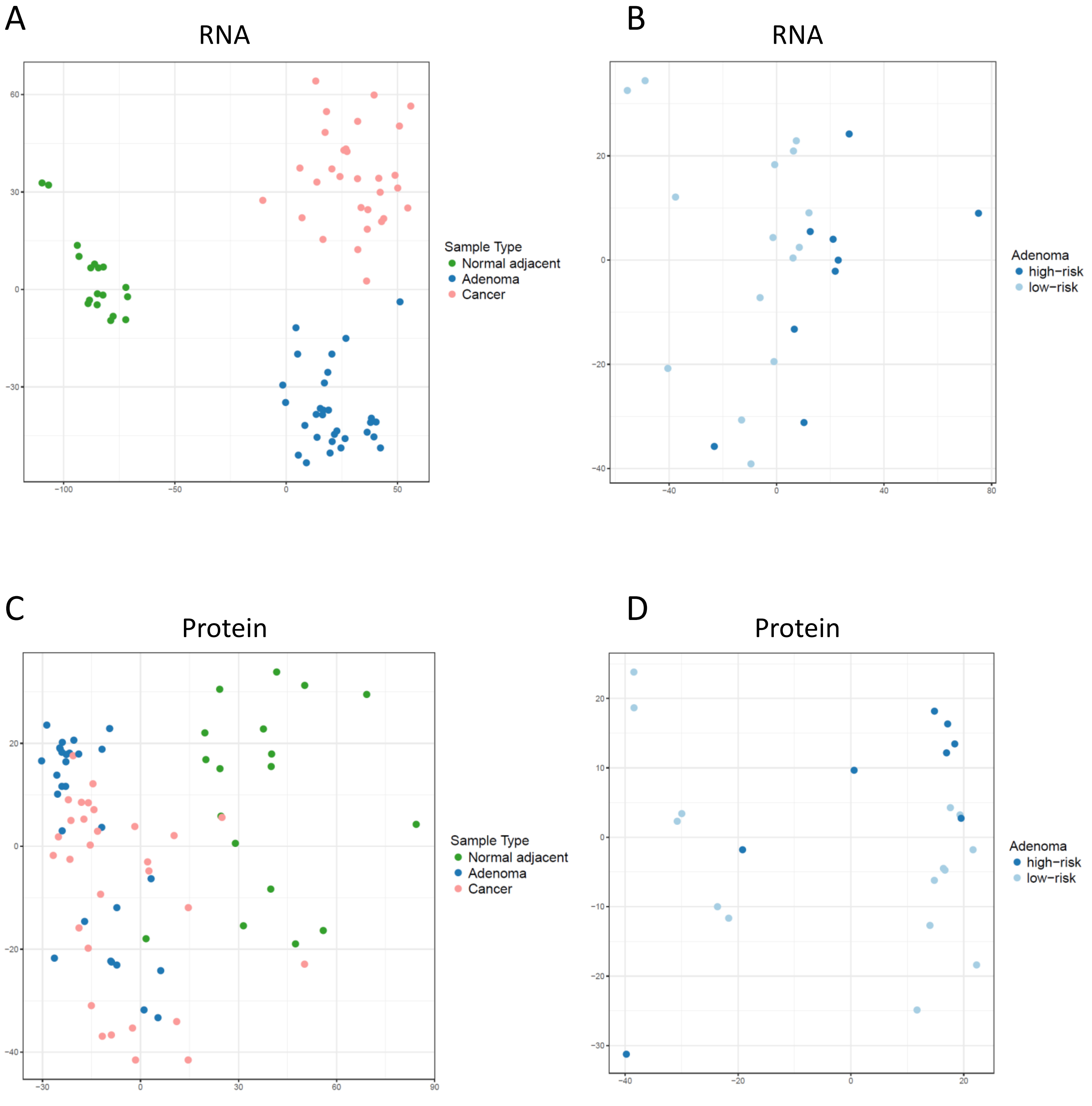

Supplementary Figure 4

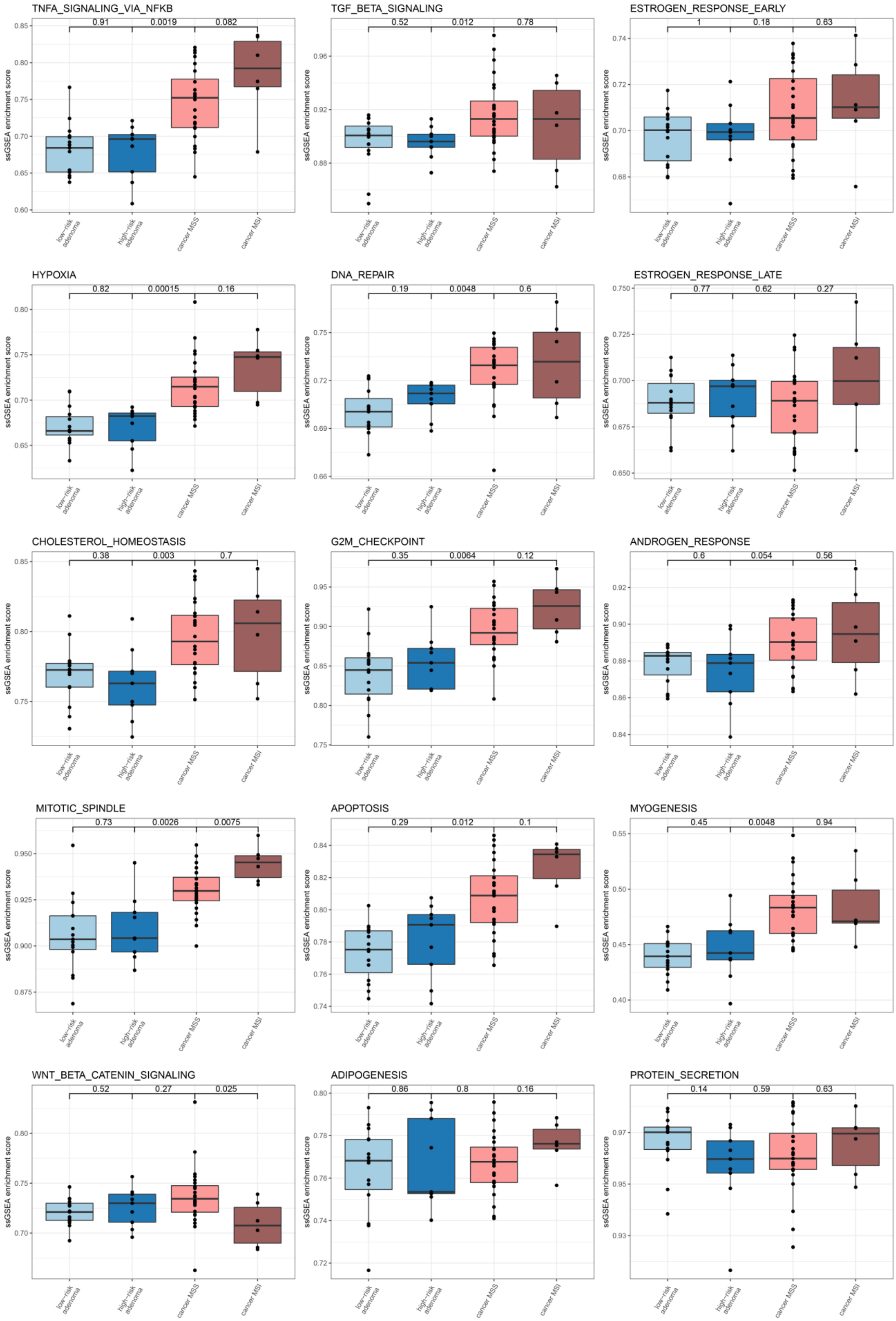

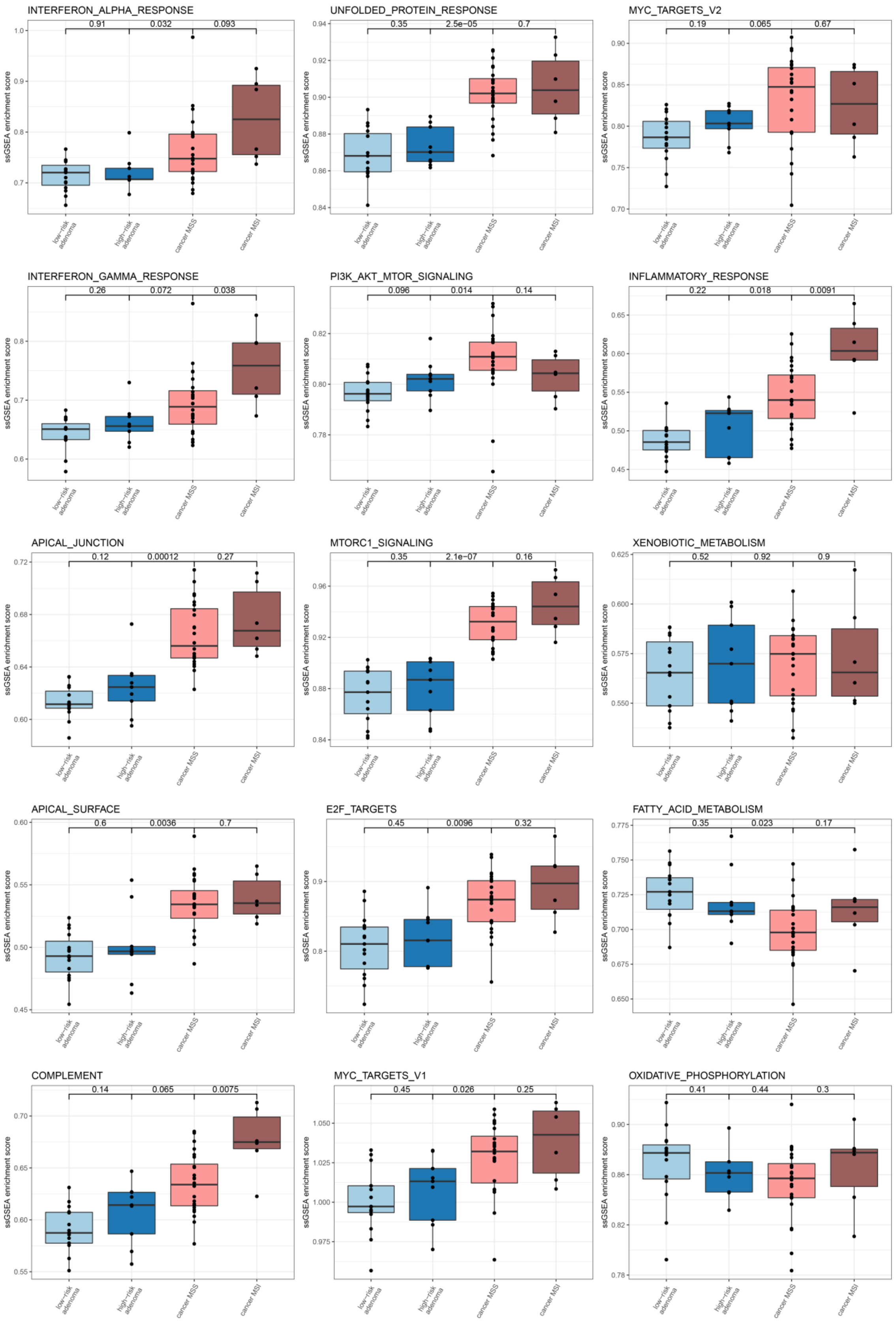

INTERFERON_ALPHA_RESPONSE
0.91 0.032 0.093
UNFOLDED_PROTEIN_RESPONSE
0.35 2.5e−05 0.7
MYC_TARGETS_V2
0.19 0.065 0.67
INTERFERON_GAMMA_RESPONSE
0.26 0.072 0.038
PI3K_AKT_MTOR_SIGNALING
0.096 0.014 0.14
INFLAMMATORY_RESPONSE
0.22 0.018 0.0091
APICAL_JUNCTION
0.12 0.00012 0.27
MTORC1_SIGNALING
0.35 2.1e−07 0.16
XENOBIOTIC_METABOLISM
0.52 0.92 0.9
APICAL_SURFACE
0.6 0.0036 0.7
E2F_TARGETS
0.45 0.0096 0.32
FATTY_ACID_METABOLISM
0.35 0.023 0.17
COMPLEMENT
0.14 0.065 0.0075
MYC_TARGETS_V1
0.45 0.026 0.25
OXIDATIVE_PHOSPHORYLATION
0.41 0.44 0.3
ssGSEA enrichment score
low−risk adenoma
high−risk adenoma
cancer MSS
cancer MSI

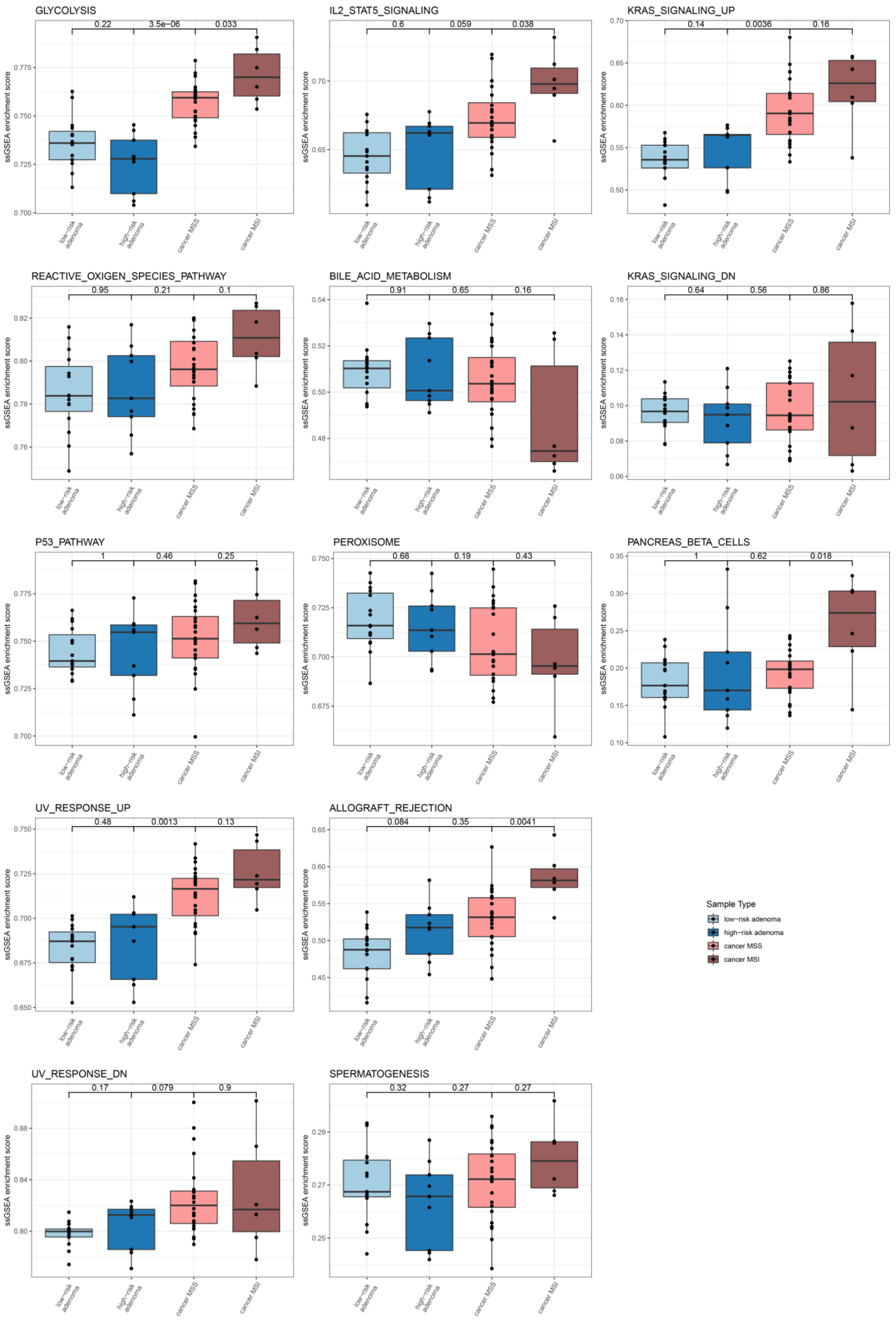
GLYCOLYSIS
0.22
3.5e−06
0.033
IL2_STAT5_SIGNALING
0.6
0.059
0.038
KRAS_SIGNALING_UP
0.14
0.0036
0.16
REACTIVE_OXIGEN_SPECIES_PATHWAY
0.95
0.21
0.1
BILE_ACID_METABOLISM
0.91
0.65
0.16
KRAS_SIGNALING_DN
0.64
0.56
0.86
P53_PATHWAY
1
0.46
0.25
PEROXISOME
0.68
0.19
0.43
PANCREAS_BETA_CELLS
1
0.62
0.018
UV_RESPONSE_UP
0.48
0.0013
0.13
ALLOGRAFT_REJECTION
0.084
0.35
0.0041
UV_RESPONSE_DN
0.17
0.079
0.9
SPERMATOGENESIS
0.32
0.27
0.27
ssGSEA enrichment score
low−risk adenoma
high−risk adenoma
cancer MSS
cancer MSI
Sample Type
low−risk adenoma
high−risk adenoma
cancer MSS
cancer MSI

Supplementary Figure 5

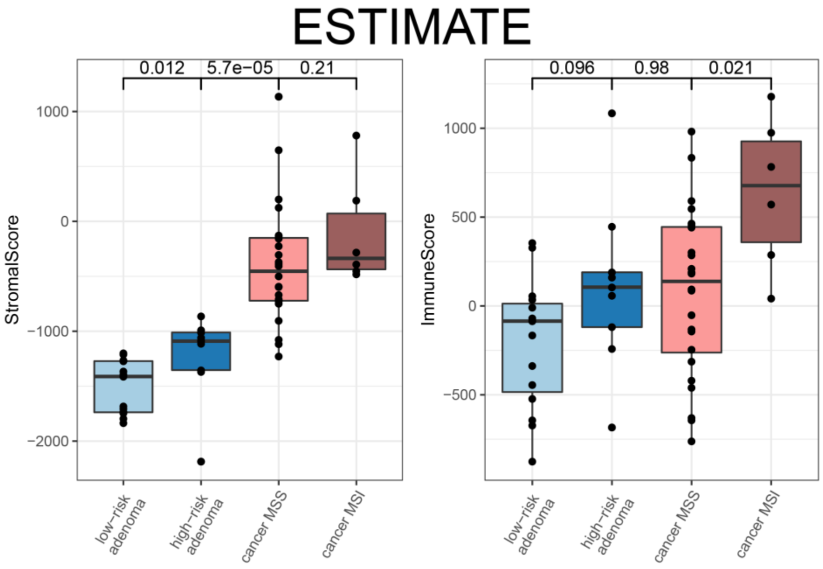

Supplementary Figure 6

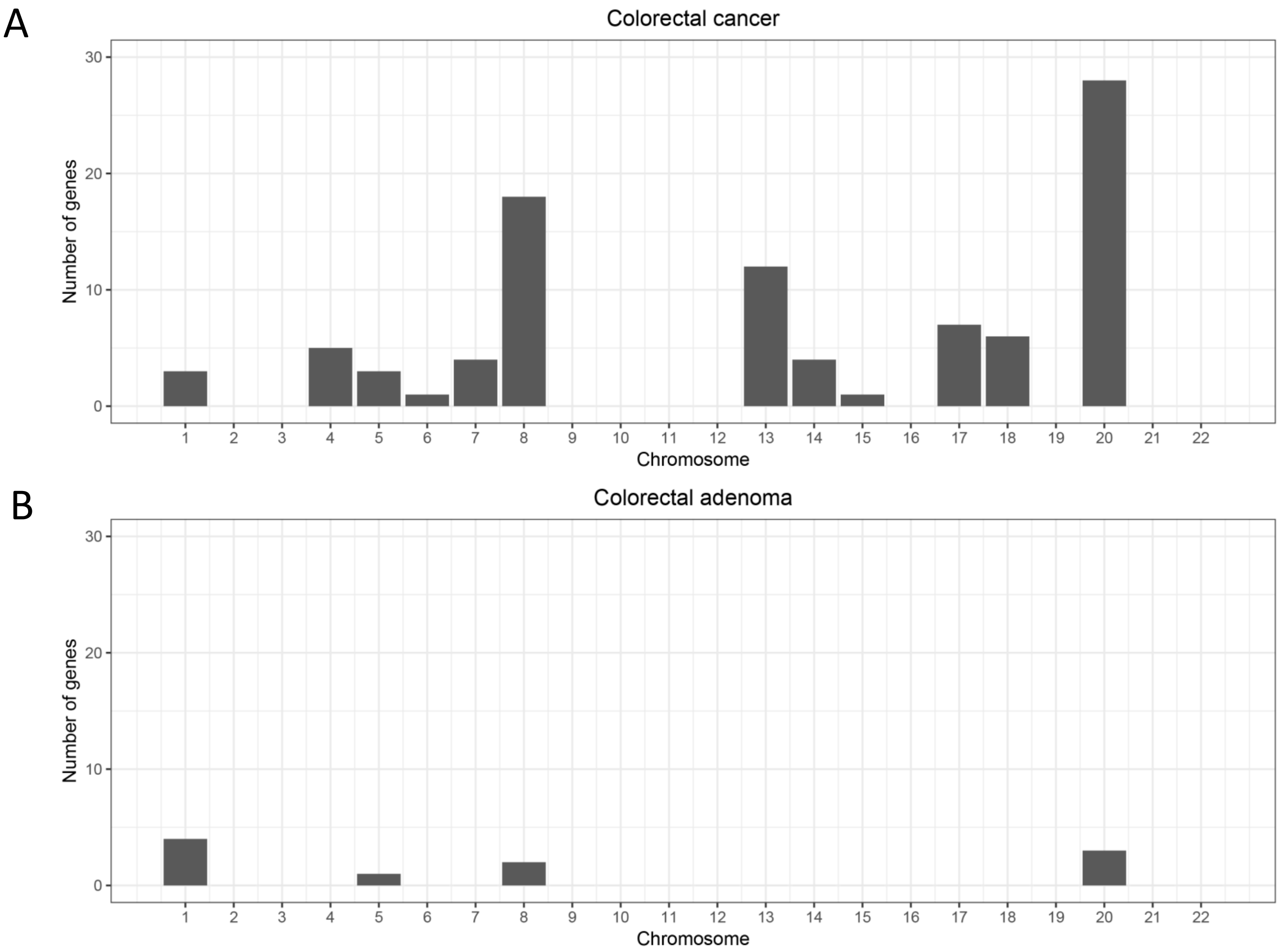

Supplementary Figure 7

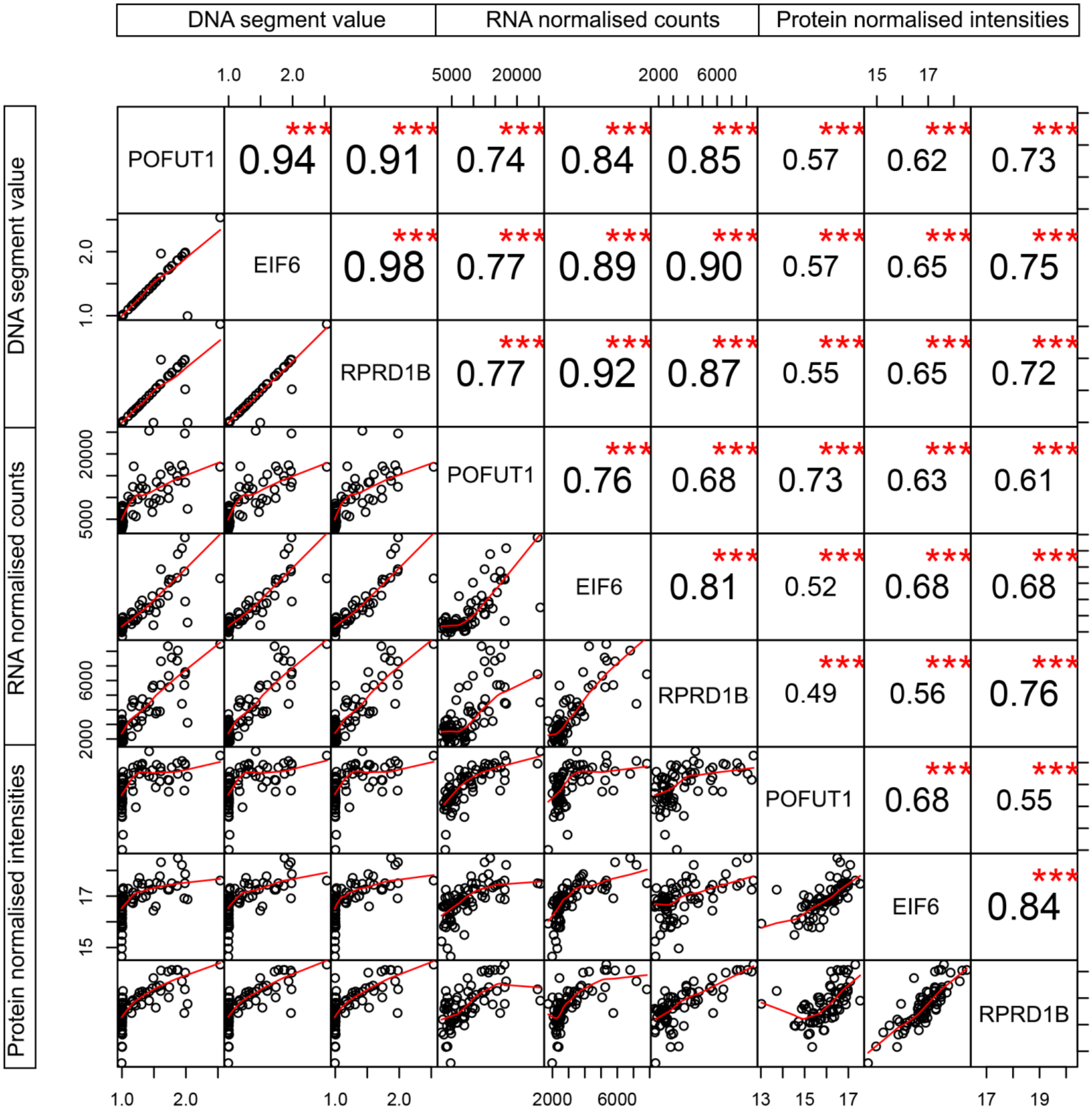

Supplementary Figure 8

A

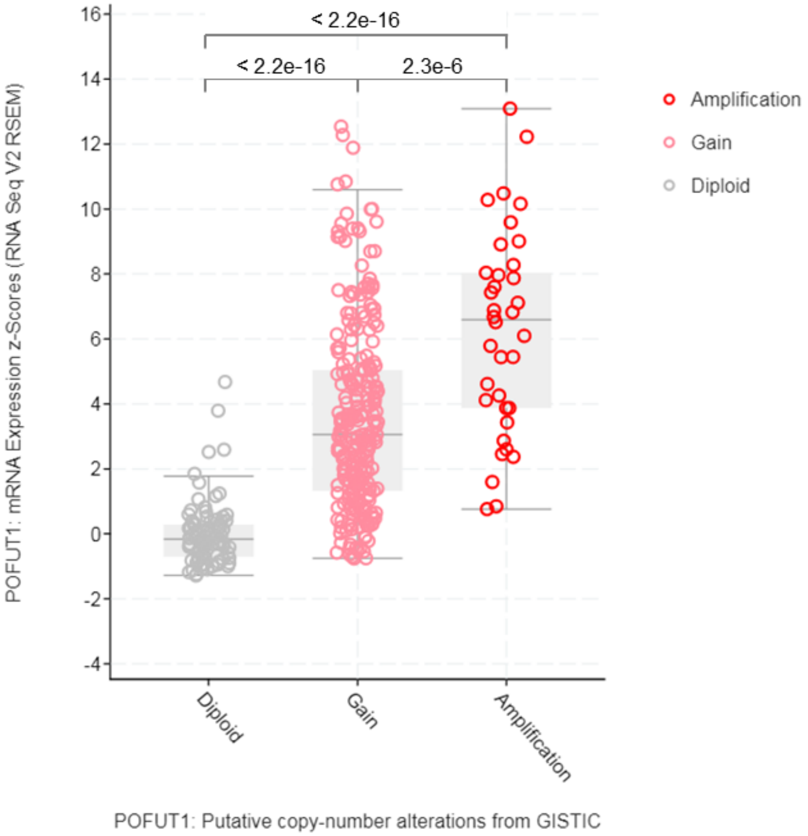

B

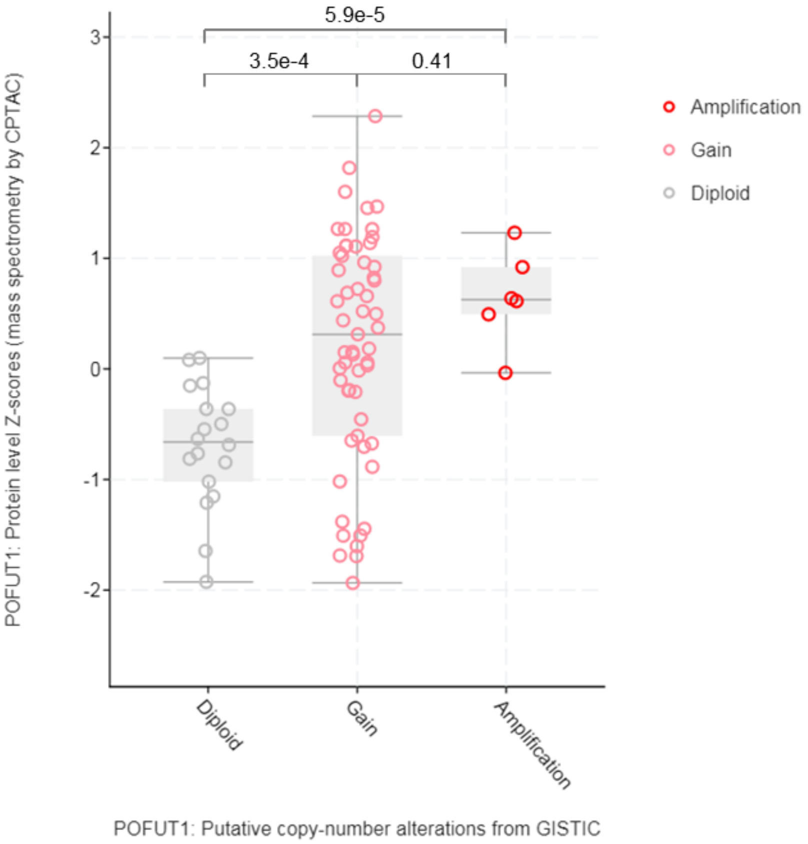

Supplementary Figure 9

A

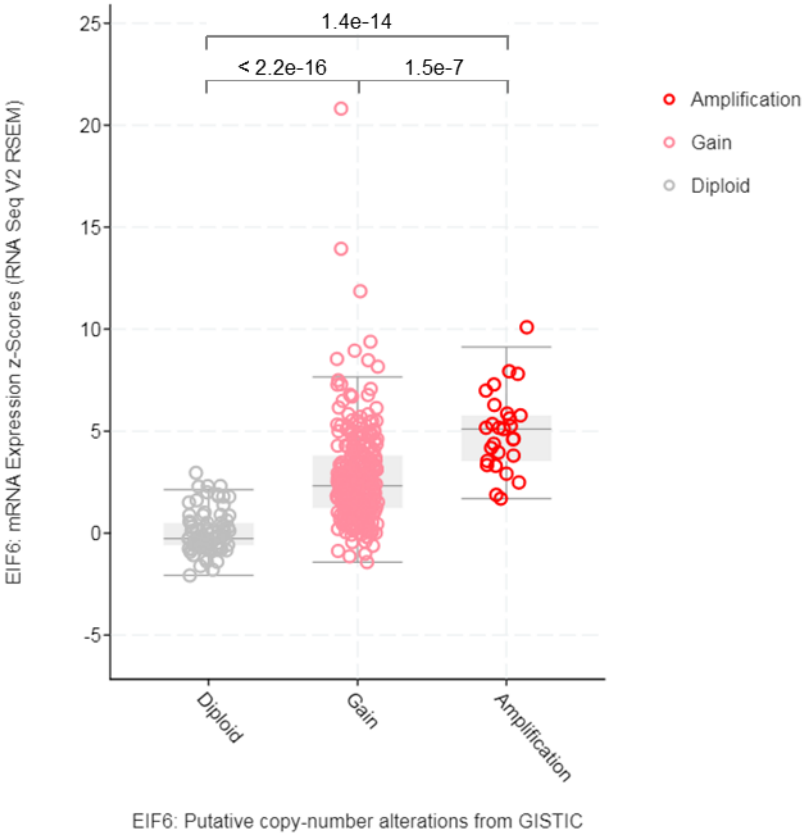

B

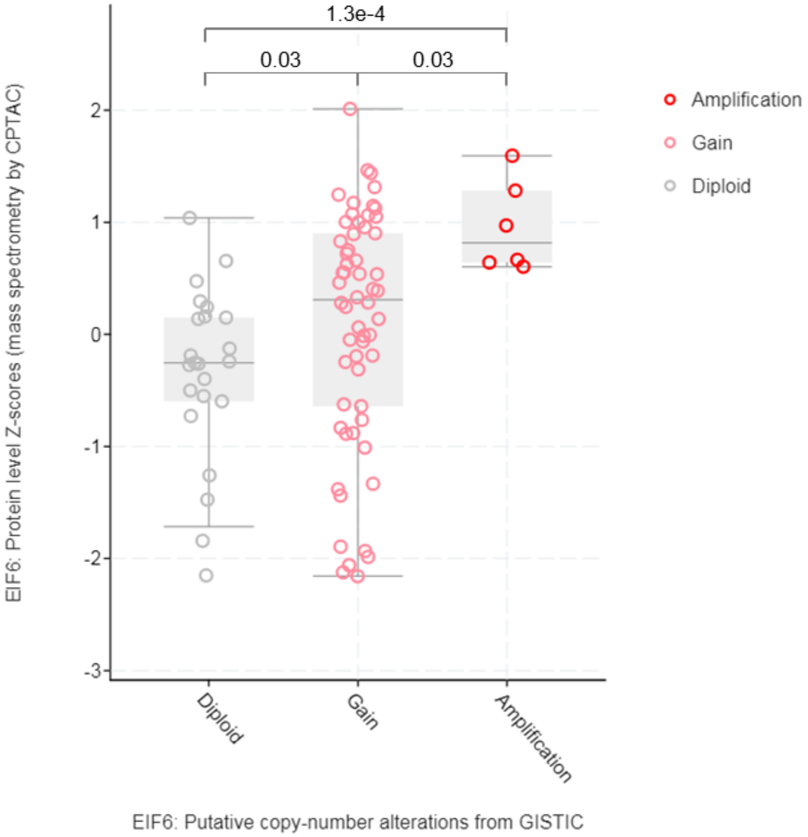

Supplementary Figure 10

A

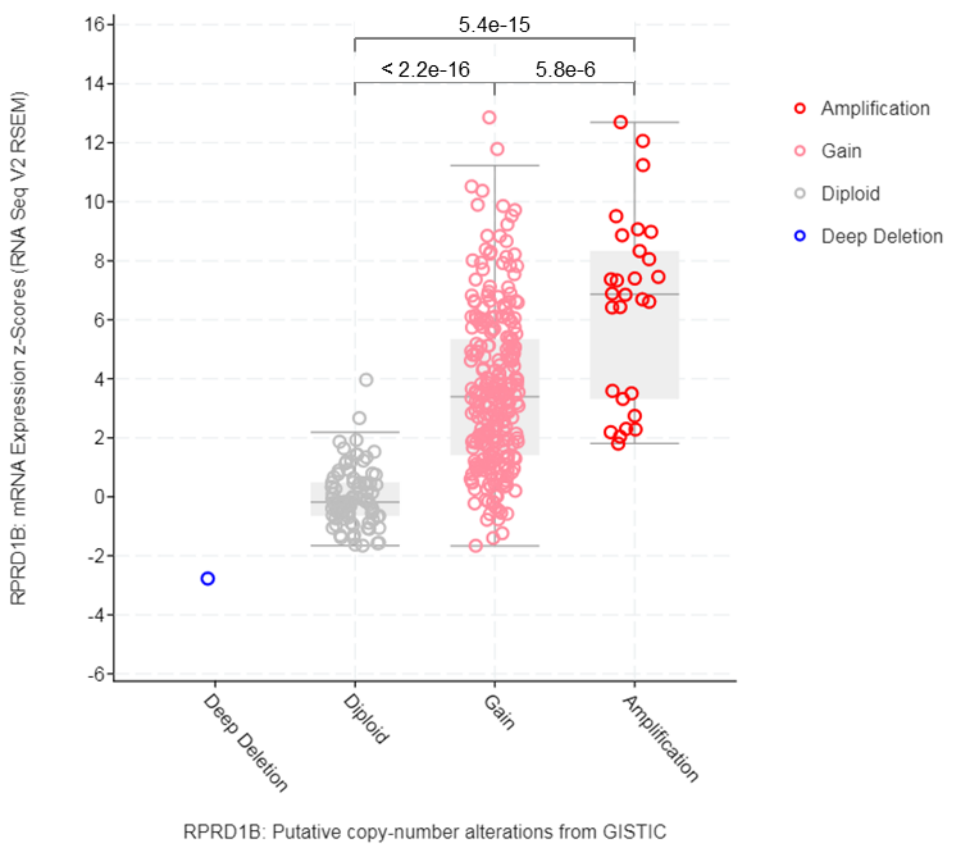

B

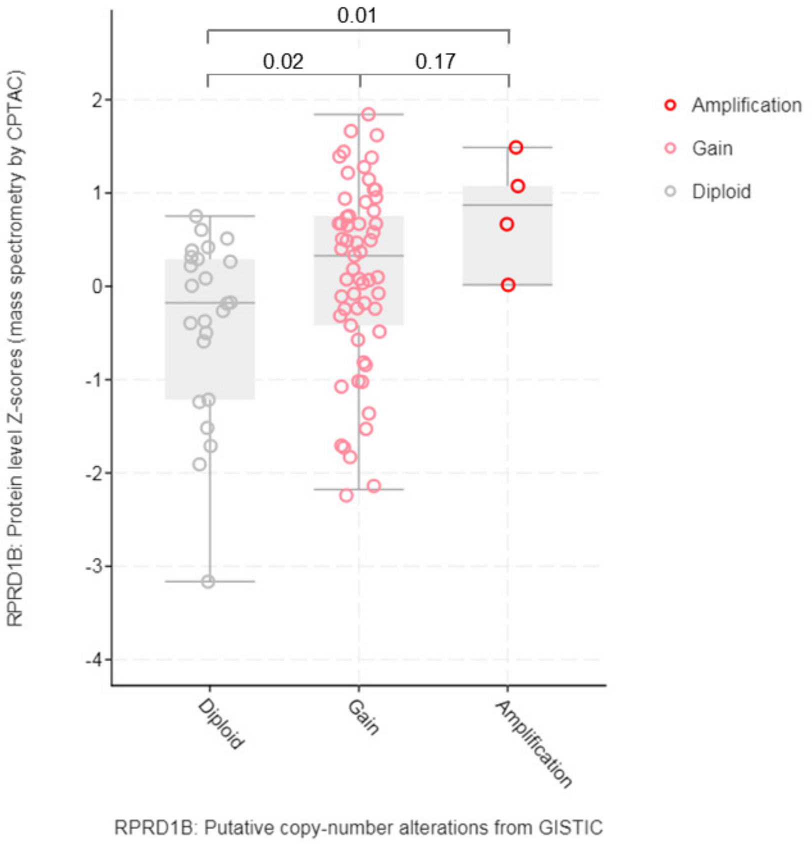

Supplementary Figure 11

A

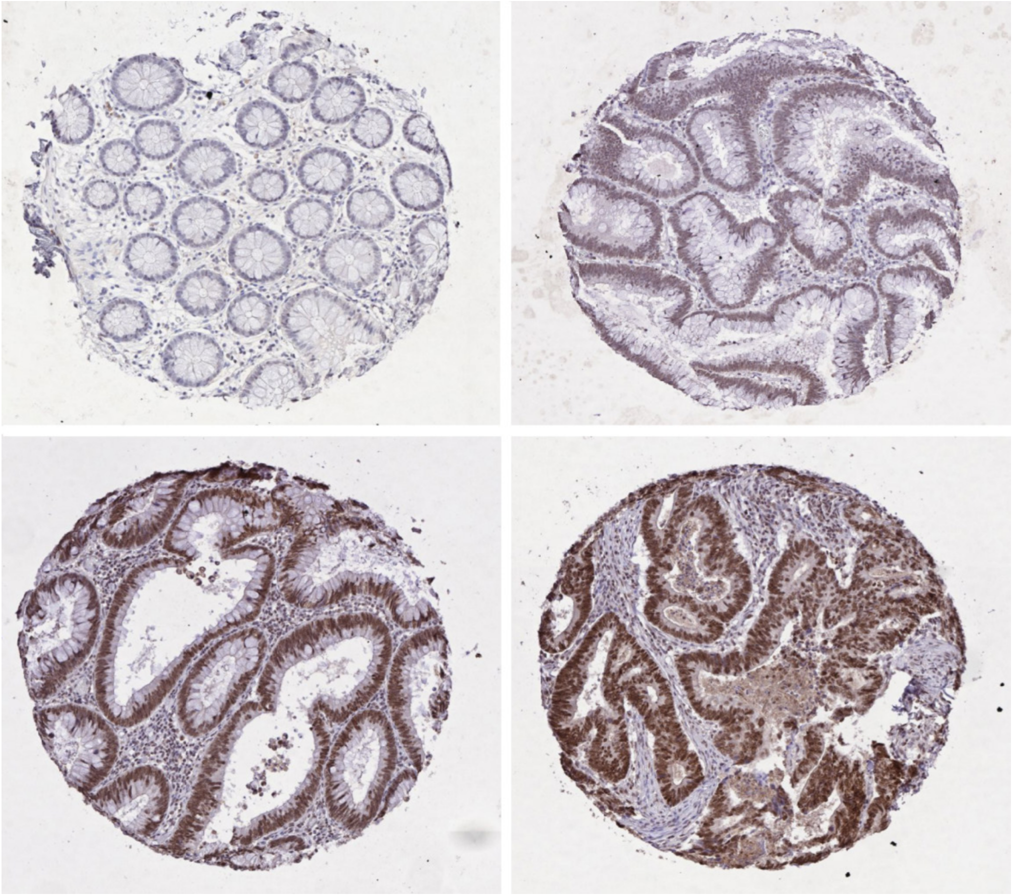

B

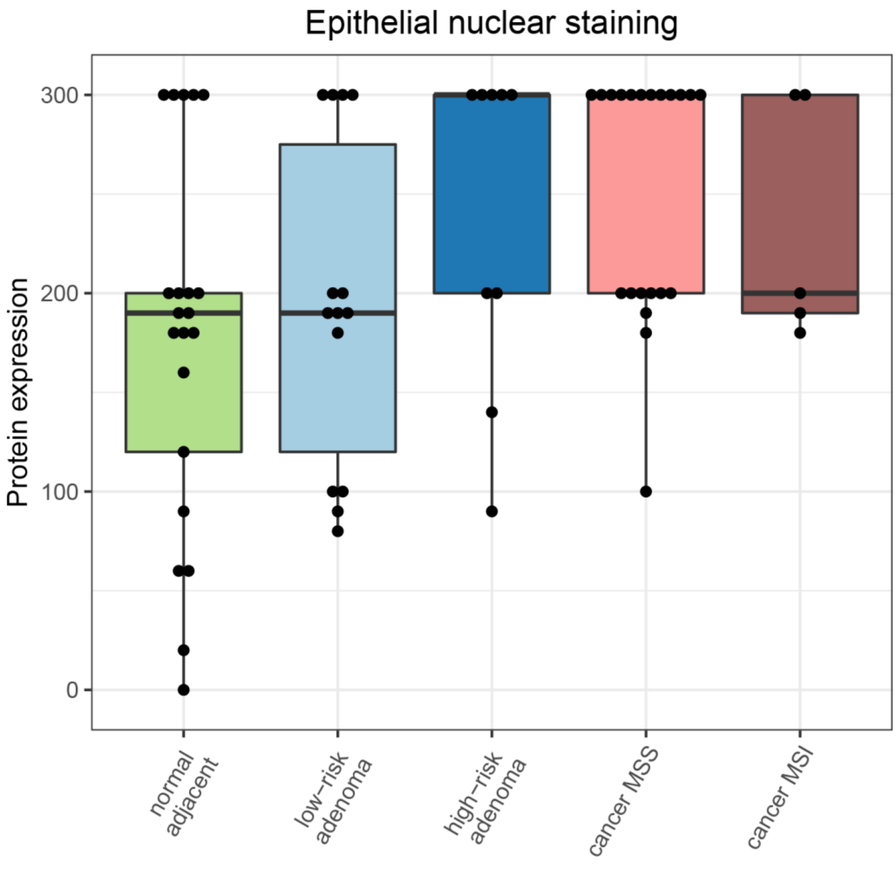

Supplement: Supplementary file 2 — Appendix S2: Supplementary Figures [file IJC-146-1979-s002.pdf]
