# Supplementary material for: Molecular characterization of colorectal adenomas reveals POFUT1 as a candidate driver of tumor progression
Source: Int J Cancer. 2019 Aug 30;146(7):1979–92. doi: 10.1002/ijc.32627 (PMC7027554; doi:10.1002/ijc.32627)
Supplement: Supplementary file 3 — Appendix S3: Supplementary Material [file IJC-146-1979-s003.doc]

# Supplementary Materials and Methods

## Sample preparation for mass spectrometry proteomics

Proteins were isolated from snap-frozen tissue samples. Twenty tissue sections of 16 μm thickness were lysed in 30µl of reducing sample buffer per mg of tissue (NuPAGE™ lithium dodecyl sulfate (LDS) Sample Buffer, supplemented with DTT 0.1M, Thermo Fisher, Bremen, Germany), thoroughly vortexed for 1 minute, heated at 70°C for 10 minutes and sonicated (3 times 20 seconds with 20 second intervals). Lysates were centrifuged for 10 minutes at 20,000 x g upon which supernatants were transferred to a new tube.

Equal amounts of proteins (~50μg) were loaded from each sample using block randomization across the three patient groups and gels (equal number of normal adjacent colon, adenoma and CRC samples in each gel). Proteins were separated on precast 4-12% gradient SDS-PAGE gels (Invitrogen, Carlsbad, USA). The gels were fixed in 50% ethanol containing 3% phosphoric acid, washed and stained overnight with Coomassie R-250. Gels were washed in ultrapure water (Merck Millipore, Billerica, MA, USA) and stored at 4°C until further processing. Each sample lane was cut from the gel as a single band and subjected to protein digestion as previously described^1, 2^. Peptides were extracted and the volume of the peptide fractions was reduced to 50 µl in a vacuum centrifuge to eliminate ACN from the solution. Peptide extracts were then desalted as an extra cleanup step using Oasis HLB cartridges (Waters Chromatography B.V, Etten-Leur, The Netherlands). Peptide eluates were dried in a vacuum centrifuge and re-dissolved in 4% acetonitrile + 0.5% Trifluoroacetic acid + 0.02% retention time peptides (iRT, Biognosys, Schlieren, Switzerland). Peptides were separated by an Ultimate 3000 nanoLC system (Dionex LC-Packings, Amsterdam, The Netherlands), equipped with a 50 cm x 75 µm ID fused silica column custom packed with 1.9 µm 120 Å ReproSil Pur C18 aqua (Dr Maisch GMBH, Ammerbuch,Entringen, Germany), as described previously^2^. After injection, peptides were trapped at 6 μl/min on a 10 mm × 100 μm ID trap column packed with 5 μm 120 Å ReproSil Pur C18 aqua at 2% buffer B (buffer A: 0.5% acetic acid in ultrapure water; buffer B: 80% ACN + 0.5% acetic acid in ultrapure water) and separated at 300 nl/min in a 10–40% buffer B linear gradient in 90 min (120 min inject-to-inject). LC-MS/MS runs were performed using block randomization across sample types, injecting in alternating order of normal colon-adenoma-cancer samples. Any instrument performance drift was equally distributed over all sample groups, thereby minimizing group bias by experiment design^3^. The mass spectrometry proteomics data have been deposited to the ProteomeXchange Consortium via the PRIDE^4^ partner repository with the accession identifier PXD012254.

## RNA sequencing data analysis

Differential gene expression analysis was performed between high-risk and low-risk adenomas using DESeq2^5^. Differentially expressed genes were obtained with the following filtering; absolute log2 fold change≥0.6 and adjusted p-value≤0.05. Regularized logarithmic transformation of expression values was performed, Euclidian distance between samples was calculated and the RNA expression data was visualized using the multidimensional scaling algorithm. Additionally, normalized counts as well as RPKMs were obtained for the whole expression matrix. Cellular decomposition analysis was performed using ESTIMATE^6^ algorithm on the RPKM expression-based matrix. Group comparison of the results was performed with the Mann-Whitney test and p-values were obtained.

## Proteomics data analysis

Separate searches were performed for differential expression analysis between high-risk and low-risk adenomas, and for obtaining protein expression matrix across all sample types (see Supplementary Table 1 for the Spectronaut parameters). Quality control was performed by comparing the total number of protein groups identified in each sample and by the multidimensional scaling algorithm on Euclidian distance of protein expression profiles. A sample was considered an outlier if its number of total protein groups was below the range of the average number of protein groups identified per sample in the whole dataset +/- 2 standard deviations of the number of protein groups identified per sample in the whole dataset, and was removed from the proteomic dataset. Protein group intensities were log2 transformed and median-centering normalization was performed. Differentially expressed proteins between high-risk and low-risk adenomas were identified using limma^7^, Benjamini-Hochberg correction was used to calculate p-value adjusted for multiple hypothesis testing (absolute log2 fold change≥0.6 and p-value≤0.05). Euclidian distance and multidimensional scaling algorithm were used for data visualization.

## Gene set enrichment analysis

Gene set enrichment analysis (GSEA) was performed after differential expression analysis between high-risk and low-risk adenomas on both RNA and protein level. Genes were ranked according to log10 transformed p-value with the sign opposite to the log2 fold change (-sign(log2FC)*log10(p-value)). The ranked list was submitted to GSEA^8^ and the collections of hallmark gene sets from Molecular Signature Database v6.0 (MSigDB) were used^9^. Significant gene sets were extracted based on an FDR threshold of ≤0.15. Single sample GSEA (ssGSEA) analysis was performed only for the RNA expression data using GSVA package^10^ on normalized counts for adenomas and cancer samples, using the collection of hallmark gene sets from MSigDB^9^. P-values for group comparison were obtained using Mann-Whitney test.

## DNA-RNA-protein correlation analysis for gene-dosage effect identification

Pairwise Spearman correlations were calculated between DNA copy number, RNA and protein expression for the genes occurring in all three datasets. The analysis was performed for the adenomas and the cancers separately. On DNA level, for each gene a segment value of its chromosomal location was assigned. On RNA level normalized counts were used and on protein level log2 transformed protein group intensities. Correlation coefficients (R_S_) and p-values were obtained. FDR was calculated with the Benjamini-Hochberg method. Significant correlations were identified, when correlation coefficient was ≥ 0.5 and FDR values were either ≤0.25 or ≤0.1 in all 3 pairwise comparisons for adenomas and cancers, respectively. Pearson correlation was also calculated between the genes of interest *EIF6, POFUT1* and *RPRD1B* for each experiment and correlation matrix was plotted using “PerformanceAnalytics” R package.

Validation of gene-dosage effect was performed using cBioPortal (<https://www.cbioportal.org/>) TCGA Provisional dataset of colorectal adenocarcinomas^11, 12^. DNA copy number (n = 616), RNA expression from RNA sequencing (n = 382) and protein expression measured by mass spectrometry proteomics (n = 90) were used. In the plot tab of cBioPortal RNA or Protein expression for each gene, POFUT1, EIF6 or RPRD1B, were plotted against the available GISTIC copy number of the same gene; deep deletion, diploid, gain, amplification. For group comparisons p-values were obtained with the use of Mann-Whitney test.

## Quantification of tumor-stroma and goblet cells

Digital images were analyzed with HALO software (v2.1, Indica Labs) to accurately determine tumor-stroma percentage. Random Forest classifier was used with the classes “epithelium”, “stroma” and “lumen”. The classifier was trained on the manually selected areas representing each class and applied on the whole tissue section. The results were evaluated by an expert pathologist. Only the stroma and epithelium areas were extracted from the results for further analysis, while the lumen area was excluded. Tumor-stroma percentage was obtained by dividing the stroma area by the sum of stroma and epithelium area.

The proportion of goblet cells in each sample was estimated by an expert pathologist, using the H&E-stained slides. The categories were few (0-20%), moderate (21-50%) and many goblet cells (more than 50%).

## Immunohistochemical staining of tissue microarrays and patient-derived colorectal adenoma organoids

Tissue microarrays (TMA) for NGS-ProToCol samples were obtained as described previously^13^. Briefly, three tissue core biopsies of 0.6 mm in diameter were punched from morphologically representative areas of the Formalin-fixed, paraffin-embedded (FFPE) donor blocks and transferred into TMA recipient paraffin blocks using 3DHISTECH TMA Master (v1.14, #dHISTECH Ltd., Budapest, Hungary). The TMA represent 29 CRCs, 9 high-risk adenomas, 14 low-risk adenomas and 24 normal adjacent colon samples. TMA sections (4 μm) were deparaffinized by xylene and rehydrated with a decreasing alcohol series. TMAs were stained using HPA antibodies directed against POFUT1 (HPA054519) and RPRD1B (HPA066290). For POFUT1 staining, antigen retrieval was performed by microwave heating in citric acid (10mM, pH6.0) and endogenous peroxidase quenching in 0.3% H_2_O_2_/methanol (30 minutes). Primary rabbit polyclonal monospecific antibody directed against human POFUT1 (1:75, 1hour, Atlas Antibodies, Stockholm, Sweden) was incubated at room temperature for 1 hour. For RPRD1B staining, antigen retrieval was performed by autoclave heating in citric acid (10mM, pH6.0) and endogenous peroxidase quenching in 0.3% H_2_O_2_/methanol (30 minutes). Primary rabbit polyclonal monospecific antibody directed against human RPRD1B (1:600, overnight, Atlas Antibodies, Stockholm, Sweden) was incubated at 4^o^C overnight. Secondary anti-rabbit antibodies (BrightVision, Immunologic, Duiven, The Netherlands) were incubated for 30 minutes at room temperature. Secondary antibodies were visualized by liquid diaminobenzidine substrate chromogen system. Incubation without primary antibody served as negative control. TMAs were scanned using Aperio AT2 Scanner (Leica Biosystems Imaging). TMA scoring was performed with the use of Slide Score ([www.slidescore.com](../../../CancerResearch/www.slidescore.com)) by an expert pathologist. For POFUT1, staining intensity (negative=0, weak=1, moderate=2 or strong=3) and percentage of the area stained (0-100%) were scored in the cytoplasm of epithelial cells within each tissue core. For RPRD1B, staining intensity (negative=0, weak=1, moderate=2 or strong=3) and percentage of the area stained (0-100%) were scored in the nuclei of epithelial cells within each tissue core. Protein expression values were obtained by multiplying staining intensity by the percentage of the area stained. Maximum values were selected from the replicate cores per sample. ROC analysis with Youden statistics were used to define the best threshold to distinguish cases from controls^14^, expression values were dichotomized based on the best threshold and p-values were obtained with Fisher exact test.

Colorectal adenoma-derived organoids were obtained from individuals participating in the Dutch colorectal cancer screening program, who underwent a colonoscopy procedure. All participants gave informed consent for the establishment of organoids and their use in molecular research. Colorectal organoids were obtained and cultured as described previously^15^ with a few modifications*.* Organoids were cultured in Matrigel® Growth Factor Reduced Basement Membrane Matrix, Phenol Red-Free (Corning) and passaged approximately once per week by enzymatic digestion using TrypLE™ Express Enzyme ((1X), phenol red., 12605010, Thermo Fisher, Bremen, Germany) or by mechanical disruption by stretched glass pipets. Droplets of organoid-containing Matrigel, dispensed in pre-warmed (37 °C) 24-wells culture plates, were overlain with complete crypt culture medium (Advanced DMEM/F12 (Invitrogen) containing growth factors 20% R-Spondin conditioned medium, 10% Noggin conditioned medium, 1× B27(Invitrogen), 1,25 mM n-Acetyl Cysteine(Sigma), 10 mM Nicotinamide(Sigma), 50 ng/ml human EGF(Peprotech), 10 nM Gastrin(Sigma), 500 nM A83-01(Tocris), 3 μM SB202190(Cayman Chemicals), 10 nM Prostaglandin E2(Sigma), and 100 μg/ml Primocin(Invitrogen), see ^15, 16^). All conditioned media were produced in-house from the cell lines 293T-HA-RspoI-Fc, HEK293-mNoggin-Fc and L-Wnt3a, as described previously ^17^. These cell lines were kindly provided by Prof. Kuo (293T-HA-RspoI-Fc) from the Leland Stanford Junior University, Palo Alto, USA and by Prof. Clevers (HEK293-mNoggin-Fc and L-Wnt3a) from the Hubrecht Institute, Utrecht, the Netherlands. Genomic DNA was isolated from cell pellets using the ReliaPrep™ gDNA Tissue Miniprep System (Z6011, Promega), according to the manufacturers protocol Standard Protocol for Animal Tissue. Low-coverage whole genome sequencing and DNA copy number aberration identification was performed, as described previously^18^.

To perform the immunohistochemical staining of the organoids, the organoid sections were prepared. For each organoid, pellets were prepared by spinning down for 5 minutes at 2000 rpm, the supernatant was discarded and the pellet was washed with cold phosphate-buffered saline (PBS). The pellet was then fixated overnight at 4^o^C with formaldehyde and afterwards mounted in a gel matrix using Cytoblock system (Thermo Fisher, Bremen, Germany). The cell suspension was transferred to a transit cassette and imbedded in paraffin. Sections were made from the paraffin blocks. Organoids were first incubated for 5 minutes in cold PBS, to remove traces of Cultrex matrix, and then stained using antibodies directed against POFUT1 (HPA054519) and RPRD1B (HPA066290) as performed for the TMAs. Scoring and statistical analysis was performed as for the TMAs.

## References

1. Piersma SR, Warmoes MO, de Wit M, de Reus I, Knol JC, Jimenez CR. Whole gel processing procedure for GeLC-MS/MS based proteomics. Proteome science 2013;11:17.

2. Piersma SR, Fiedler U, Span S, Lingnau A, Pham TV, Hoffmann S, Kubbutat MH, Jimenez CR. Workflow comparison for label-free, quantitative secretome proteomics for cancer biomarker discovery: method evaluation, differential analysis, and verification in serum. Journal of proteome research 2010;9:1913-22.

3. Pham TV, Piersma SR, Oudgenoeg G, Jimenez CR. Label-free mass spectrometry-based proteomics for biomarker discovery and validation. Expert review of molecular diagnostics 2012;12:343-59.

4. Vizcaino JA, Csordas A, del-Toro N, Dianes JA, Griss J, Lavidas I, Mayer G, Perez-Riverol Y, Reisinger F, Ternent T, Xu QW, Wang R, et al. 2016 update of the PRIDE database and its related tools. Nucleic acids research 2016;44:D447-56.

5. Love MI, Huber W, Anders S. Moderated estimation of fold change and dispersion for RNA-seq data with DESeq2. Genome biology 2014;15:550.

6. Yoshihara K, Shahmoradgoli M, Martínez E, Vegesna R, Kim H, Torres-Garcia W, Treviño V, Shen H, Laird PW, Levine DA, Carter SL, Getz G, et al. Inferring tumour purity and stromal and immune cell admixture from expression data. Nature Communications 2013;4:2612.

7. Ritchie ME, Phipson B, Wu D, Hu Y, Law CW, Shi W, Smyth GK. limma powers differential expression analyses for RNA-sequencing and microarray studies. Nucleic acids research 2015;43:e47.

8. Subramanian A, Tamayo P, Mootha VK, Mukherjee S, Ebert BL, Gillette MA, Paulovich A, Pomeroy SL, Golub TR, Lander ES, Mesirov JP. Gene set enrichment analysis: a knowledge-based approach for interpreting genome-wide expression profiles. Proceedings of the National Academy of Sciences of the United States of America 2005;102:15545-50.

9. Liberzon A, Birger C, Thorvaldsdóttir H, Ghandi M, Mesirov Jill P, Tamayo P. The Molecular Signatures Database Hallmark Gene Set Collection. Cell Systems 2015;1:417-25.

10. Hänzelmann S, Castelo R, Guinney J. GSVA: gene set variation analysis for microarray and RNA-Seq data. BMC Bioinformatics 2013;14:7.

11. Cerami E, Gao J, Dogrusoz U, Gross BE, Sumer SO, Aksoy BA, Jacobsen A, Byrne CJ, Heuer ML, Larsson E, Antipin Y, Reva B, et al. The cBio cancer genomics portal: an open platform for exploring multidimensional cancer genomics data. Cancer Discov 2012;2:401-4.

12. Gao J, Aksoy BA, Dogrusoz U, Dresdner G, Gross B, Sumer SO, Sun Y, Jacobsen A, Sinha R, Larsson E, Cerami E, Sander C, et al. Integrative analysis of complex cancer genomics and clinical profiles using the cBioPortal. Sci Signal 2013;6:pl1.

13. Goos JA, Coupe VM, Diosdado B, Delis-Van Diemen PM, Karga C, Belien JA, Carvalho B, van den Tol MP, Verheul HM, Geldof AA, Meijer GA, Hoekstra OS, et al. Aurora kinase A (AURKA) expression in colorectal cancer liver metastasis is associated with poor prognosis. British journal of cancer 2013;109:2445-52.

14. Robin X, Turck N, Hainard A, Tiberti N, Lisacek F, Sanchez J-C, Müller M. pROC: an open-source package for R and S+ to analyze and compare ROC curves. BMC Bioinformatics 2011;12:77.

15. Sato T, Vries RG, Snippert HJ, van de Wetering M, Barker N, Stange DE, van Es JH, Abo A, Kujala P, Peters PJ, Clevers H. Single Lgr5 stem cells build crypt-villus structures in vitro without a mesenchymal niche. Nature 2009;459:262-5.

16. van de Wetering M, Francies Hayley E, Francis Joshua M, Bounova G, Iorio F, Pronk A, van Houdt W, van Gorp J, Taylor-Weiner A, Kester L, McLaren-Douglas A, Blokker J, et al. Prospective Derivation of a Living Organoid Biobank of Colorectal Cancer Patients. Cell 2015;161:933-45.

17. Jung P, Sato T, Merlos-Suarez A, Barriga FM, Iglesias M, Rossell D, Auer H, Gallardo M, Blasco MA, Sancho E, Clevers H, Batlle E. Isolation and in vitro expansion of human colonic stem cells. Nature medicine 2011;17:1225-7.

18. Carvalho B, Diosdado B, Terhaar Sive Droste JS, Bolijn AS, Komor MA, de Wit M, Bosch LJW, van Burink M, Dekker E, Kuipers EJ, Coupe VMH, van Grieken NCT, et al. Evaluation of Cancer-Associated DNA Copy Number Events in Colorectal (Advanced) Adenomas. Cancer prevention research (Philadelphia, Pa.) 2018;11:403-12.
